# Variation of Passive Biomechanical Properties of the Small Intestine along Its Length: Microstructure-Based Characterization

**DOI:** 10.3390/bioengineering8030032

**Published:** 2021-02-26

**Authors:** Dimitrios P. Sokolis

**Affiliations:** Laboratory of Biomechanics, Center of Clinical, Experimental Surgery, and Translational Research, Biomedical Research Foundation of the Academy of Athens, 115 27 Athens, Greece; dsokolis@bioacademy.gr or dimitrissokolis@ath.forthnet.gr

**Keywords:** microstructure-based material formulations, small intestinal segments, collagen structure, fiber families, layer-specific thickness, passive properties

## Abstract

Multiaxial testing of the small intestinal wall is critical for understanding its biomechanical properties and defining material models, but limited data and material models are available. The aim of the present study was to develop a microstructure-based material model for the small intestine and test whether there was a significant variation in the passive biomechanical properties along the length of the organ. Rat tissue was cut into eight segments that underwent inflation/extension testing, and their nonlinearly hyper-elastic and anisotropic response was characterized by a fiber-reinforced model. Extensive parametric analysis showed a non-significant contribution to the model of the isotropic matrix and circumferential-fiber family, leading also to severe over-parameterization. Such issues were not apparent with the reduced neo-Hookean and (axial and diagonal)-fiber family model, that provided equally accurate fitting results. Absence from the model of either the axial or diagonal-fiber families led to ill representations of the force- and pressure-diameter data, respectively. The primary direction of anisotropy, designated by the estimated orientation angle of diagonal-fiber families, was about 35° to the axial direction, corroborating prior microscopic observations of submucosal collagen-fiber orientation. The estimated model parameters varied across and within the duodenum, jejunum, and ileum, corroborating histologically assessed segmental differences in layer thicknesses.

## 1. Introduction

The small intestine is the longest organ in the digestive tract, lying between the stomach and large intestine. It is the site of absorption of nutrients from food and responsible for maintaining water and electrolyte balance, providing an immunologic barrier, and endocrine secretion [1]. Knowledge of its biomechanical characteristics is vital in appreciating the transport and other small intestinal functions in health and disease [2]. This tissue is a layered structure composed of complicated formations of collagen fibers and cells, conferring to it its highly deformable, residually stressed, anisotropic, and non-linearly pseudo-elastic characteristics. Major developments, originating for a large part from Gregersen and coworkers, have improved our understanding of these characteristics [3,4,5,6]. Nevertheless, no microstructure-based material formulation for small intestinal wall tissue has been developed, although the consensus today is the implementation of such approaches because the material response of soft biologic tissues has an evident structural basis and the physical meaning of phenomenological model parameters remains ambiguous. Our group [7] and others, e.g., [8,9,10], have developed microstructure-based formulations for the large intestine, accounting for anisotropy via fiber families with discrete orientation angles.

The present study developed a microstructure-based formulation for the small intestine and detected biomechanical property variations along its length. Histologic justification was supplied from observations on different intestinal segments. The muscle component and ground matrix of the wall responsible for the low-stress response was accounted for by utilizing a neo-Hookean term. Axially aligned collagen fibers conferred to the tissue its axial stiffness at physiologic and high stresses, while circumferentially and diagonally aligned collagen fibers conferred circumferential tissue stiffness. Variants of the four-fiber model with fewer parameters were explicitly put to test. Parameter values were determined by fitting passive quasi-static inflation/extension data covering and exceeding the physiologic load range. The comprehensive eight-parameter model and a six-parameter alternative with diagonal- and axial-fiber families produced substantially better data representations for all small intestinal segments than our past attempts with phenomenological models [11]. Of note, the reduced microstructure-based model dispensed quantitative interpretations in agreement with histologically observed collagen fiber orientations and layer-specific thickness data.

## 2. Materials and Methods

### 2.1. Biologic Tissue and Biomechanical Testing

Microstructure-based formulations for the small intestine were studied on experimental data borrowed from our previous work [11]. In addition to the 40 specimens that were reported there, further testing was conducted, bringing the total number of specimens to 48. Details about the material used and our inflation/extension testing methods were given in our previous article. Briefly described, tubular specimens with a length of 3 cm from eight distinct small intestinal segments were used to identify segmental differences. The animals from which the specimens were harvested were healthy male Wistar rats with an average age of 12 months that had been used in other unrelated experiments. The small intestine was removed immediately after euthanasia, trimmed of adherent tissues, and cut into proximal and distal duodenum, as well as into proximal, middle, and distal jejunum and into proximal, middle, and distal ileum. The location of each segment along the length of the small intestine was decided using the pylorus, the ligament of Treitz, and the ileocecal valve as reference points. The mesenterium was dissected, and the contents were taken away by gently flushing through the lumen using saline. The specimens were stored in a calcium-free Krebs solution and refrigerated until testing, while pieces of tissue adjacent to them were stored for histomorphometric evaluation. All the biomechanical tests were concluded within 8 h of tissue harvesting.

**Figure 1:**
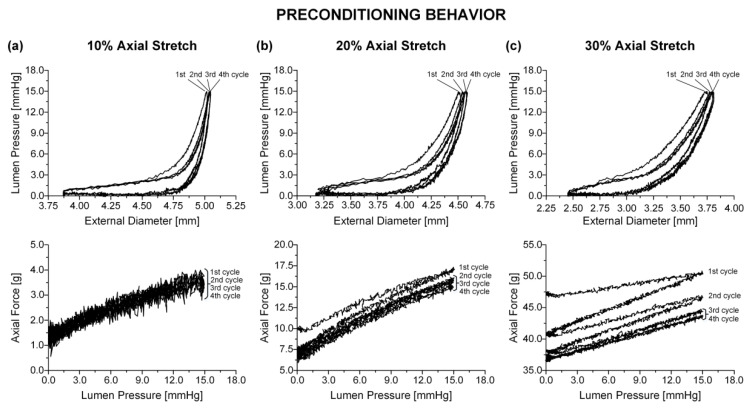
Online record of preconditioning for a distal duodenal specimen at (**a**) $\lambda_{z}=$ 1.1, (**b**) $\lambda_{z}=$ 1.2, and (**c**) $\lambda_{z}=$ 1.3. The 2nd, 3rd, and 4th pressure-diameter cycles overlapped and were enveloped by the larger 1st cycle. Τhe area enclosed by the 1st and 2nd force-pressure cycles increased with increasing $\lambda_{z}$, with all cycles overlapping at $\lambda_{z}=$ 1.1 and the 3rd and 4th cycles being very slim and nearly overlapping at $\lambda_{z}=$ 1.2 and 1.3.

Biaxial biomechanical data were collected by pressurizing the specimens quasi-statically (0.15 mmHg/s) from 0 to 15 mmHg for a minimum of four axial stretches (1.0, 1.1, 1.2, 1.3), while immersed in an oxygenated (5% CO_2_ in O_2_) calcium-free Krebs solution (37 °C); EGTA was added to the tissue bath to abolish smooth muscle tone. The pressure range in our experiments was selected to encompass sub-physiologic (0–4 mmHg), physiologic (4–12 mmHg), and supra-physiologic pressures (12–15 mmHg) [2], while allowing for a suitable deformation range. A suitable range of axial stretches encompassing the physiologic condition (~1.1) was also applied to the specimens. Preconditioning was achieved by applying four pressurization cycles at each axial stretch (Figure 1). The inflating portion of a fifth stable cycle over the same pressure range was used for data analysis. The effect of the pressurization rate was previously examined [11] by varying the driving speed of the syringe pump. Little tissue stiffening was observed when increasing the pressurization rate between 0.1 and 1.5 mmHg/s, that is, less than 5% rise in both principal stresses at the maximum strain levels. These findings, together with the slim hysteresis after three inflation/extension cycles, qualified the selected pressurization rate as quasi-static and indicated that pseudo-elasticity was a valid approximation for the small intestinal tissue. A pressure transducer (BLPR; World Precision Instruments, Hertfordshire, UK) measured the lumen pressure, a force transducer (Fort 100; World Precision Instruments, Hertfordshire, UK) the axial force, and a laser micrometer (LS-3100; Keyence Corp, Osaka, Japan) the external diameter. The device and peripheral components were controlled with a computer running a LabView program (v7.1; National Instruments, Austin, TX, USA). After testing, four rings were removed from the midpoint of the small intestinal specimens for the determination of the no-load and zero-stress states.

### 2.2. Histomorphometric Evaluation

Unloaded small intestinal specimens from the eight segments, 3 to 4 cm in length, were fixed in 10% neutral buffered formalin over 24 h (HT501128; Sigma Aldrich, St. Louis, MO, USA), dehydrated in an ascending series of ethanol (50%, 80%, 95%, and 100%), diaphanized in xylol, and embedded in paraffin wax (Paramat Extra, 361334C; VWR International, Radnor, PE, USA). Five-μm thick circumferential and axial sections were cut on a microtome (Leica RM 2125; Leica, Wetzlar, Germany) and stained with hematoxylin-eosin (Hematoxylin Harris, 3519455; VWR and Eosin solution aqueous, HT110232; Sigma-Aldrich) staining cell nuclei black, orcein (101450130; Sigma-Aldrich) staining elastin fibers dark brown, and Sirius red (43665; Fluka, Buchs, Switzerland) staining collagen fibers red, respectively. Intestinal wall morphometric analysis was carried out on images captured using a digital camera (DFV500; Leica Microsystems GmbH) and a Leica DMLS2 optical microscope. Images covering the entire intestinal perimeter in circumferential hematoxylin-eosin stained sections were captured with a 4× objective lens to measure the thickness of the mucosal, submucosal, muscular, and serosal layers. Ten thickness measurements evenly distributed over the perimeter of each specimen and three serial sections were analyzed to obtain a single value for every specimen using the Image-Pro Plus v6.0 software (Media Cybernetics Inc, Rockville, MD, USA). The orientation of collagen fibers in the various layers was inspected on images of Sirius red stained sections.

### 2.3. Microstructure-Based Material Models

The small intestine exhibited nonlinear, anisotropic, and pseudo-elastic wall properties, along with residual strains and large deformability, as is the case with the other organs of the gastrointestinal tract [2]. Incompressibility was assumed, but it has not been previously demonstrated for the small intestine. The general framework for finite inflation and axial extension of a residually stressed, thick-walled cylinder is presented in standard textbooks of biomechanics, e.g., [12]. The use of the thick-walled theory was justified by the no-load wall thickness-to-diameter ratio, that was higher than 0.25 at all small intestinal segments in our previous work [11]; the considerable thickness of the small intestinal wall was also apparent in the histologic sections of duodenal, jejunal, and ileal specimens.

Unlike the parameters of phenomenological models, the parameters of microstructure-based models admit a straightforward physical interpretation, so that the latter models may serve as more efficient constitutive descriptors for computational simulations. The microstructure-based material model for the small intestine was of the type used in our previous large intestinal study [7]. It consisted of an amorphous matrix and locally parallel fiber families—two diagonal- (denoted by superscripts *d* and *d′*), an axial- (denoted by superscript *a*), and a circumferential-fiber family (denoted by superscript *c*)—expressed as:

(1) $$W=\frac{\mu }{2}\left(I_{1}-3\right)+\sum_{j=d,d^{’},a,c}\frac{k_{1}^{j}}{4k_{2}^{j}}\left\{exp\left[k_{2}^{j}{\left(\lambda_{j}^{2}-1\right)}^{2}\right]-1\right\},$$

where W was the resultant model for the entire wall under passive conditions, i.e., negligible active muscle tone. Note that the first term on the right-hand side of Equation (1), i.e., the neo-Hookean model, contributed purely isotropically to the biomechanical response of the small intestinal wall via parameter *μ* with stress units and the first invariant $I_{1}=trC$ of the right Cauchy–Green strain tensor **C**. Given the large amount of smooth muscle cells in the small intestinal wall, especially in the thick muscle layer, their passive contribution, along with the ground matrix within which the fibrous elements and smooth muscle cells reside, were assumed to determine the neo-Hookean model. The exponential terms in Equation (1) accounted for the anisotropic characteristics of collagen fibers in the tissue. Although radial fibers, caused by the presence of mucosal foldings, were histologically observed in the no-load state of the tissue, they were not included in the model for simplicity. We assumed that under physiologic pressures, when collagen fibers are engaged, the small intestine had attained an axisymmetric cylindrical geometry internally, without foldings, and collagen fibers of the mucosa no longer occurred in the radial direction.

Model parameters $k_{1}^{j}$, $j=d,\;d^{’},a,\;c$ specified stiffness independent of deformation and were with stress units, whereas the unit-less parameters $k_{2}^{j}$ specified the progressive stiffening of the fiber families with increasing deformation. $\lambda^{j}=\sqrt{n^{j}\cdot Cn^{j}}$ were the stretches of the fiber families, whose unit vectors $n^{j}$ subtended angles $a^{j}$ with respect to the axial direction in the zero-stress state. The contribution of the fiber families was vanishing under compressive stretches, $\lambda_{j}<1$. The diagonal fibers were symmetric, $a^{d}=-\alpha^{d^{’}}=a_{0}$, with equal parameters, $k_{1}^{d}=k_{1}^{d^{’}}$ and $k_{2}^{d}=k_{2}^{d^{’}}$. Even though the model considered a homogeneous wall, the transmural variation of components in the different layers was implicitly expressed by the several fiber families.

With the incompressibility constraint enforced via a Lagrange multiplier p, the principal Cauchy stresses were established as:

(2) $$\sigma_{\theta }=-p+\lambda_{\theta }^{2}\frac{\partial W}{\partial E_{\theta }},\;\sigma_{z}=-p+\lambda_{z}^{2}\frac{\partial W}{\partial E_{z}},\;\sigma_{r}=-p+\lambda_{r}^{2}\frac{\partial W}{\partial E_{r}}.$$

Lumen pressure P and axial force F were acquired by considering the equilibrium equations along the radial *r*- and axial *z*-directions:

(3) $$P=\int_{r_{i}}^{r_{e}}\frac{\sigma_{\theta }-\sigma_{r}}{r}dr,\;F=\pi \int_{r_{i}}^{r_{e}}\left[2\left(\sigma_{z}-\sigma_{r}\right)-\left(\sigma_{\theta }-\sigma_{r}\right)\right]rdr.$$

Inserting Equation (2) into Equation (3), the pressure-diameter and force-diameter relations were recovered.

### 2.4. Parameter Estimation

The Nelder–Mead optimization algorithm in MicroCal Origin v9.0 (OriginLab Corp, Northampton, MA, USA) was used to determine the model parameters within Equation (1) by fitting Equations (2) and (3) to the data for each specimen. The residual sum of squares (*RSS*) was minimized:

(4) $$RSS=\sum_{m,n}\left[{\left(\frac{P_{mn}^{exp}-P_{mn}^{mod}}{P_{mn}^{exp}}\right)}^{2}+{\left(\frac{F_{mn}^{exp}-F_{mn}^{mod}}{F_{mn}^{exp}}\right)}^{2}\right],$$

where $P_{mn}^{exp}$ and $F_{mn}^{exp}$ were the experimentally recorded lumen pressure and axial force, and $P_{mn}^{mod}$ and $F_{mn}^{mod}$ were the modeled lumen pressure and axial force; the recorded external diameters served as independent variables. To assure consistency from one specimen to another, given that the jejunal and ileal specimens were submitted to additional axial stretches compared to the duodenal specimens, that seldom reached the 1.4 level, data assembled from the three axial stretch ratios to which all the specimens were submitted were used to determine the model parameters. Every 0.025 mmHg data point from 1 to 15 mmHg was selected, and the three datasets for the 1.1–1.3 axial stretches were combined, resulting in 3600 data points. The data of the 1.0 axial stretch, associated with buckling and negative axial forces, were intentionally omitted from the minimization procedure. Thermodynamic inequalities, ensuring model convexity and physically realistic parameter values, prescribed a zero lower limit for all parameters and a 1.57 rad upper limit for $a_{0}$ due to the symmetry of the diagonal-fiber families. The minimization procedure was repeated for a wide range of initial parameter values, $\left\{\mu ,k_{1}^{j}\right\}\in \left[0.1,10^{5}\right]$ Pa and $k_{2}^{j}\in \left[10^{-3},10^{3}\right]$, to ensure global minima. $a_{0}\in \left[0.349,\;1.211\right]$ rad confined the diagonal fibers from obtaining an essentially circumferential or axial orientation. Instead of prescribing the fiber orientation angle from quantitative analysis of histologic images, it was taken as another model parameter to be determined from the experimental data. The goodness of fit was estimated by the determination coefficient $R^{2}$ and root-mean-square error $\varepsilon =\sqrt{\chi^{2}}$, where $\chi^{2}$ was *RSS* divided by the total number of experimental points minus the number of parameters.

The smallest parameter number should generally be utilized to avoid numerical instability problems with computational implementations in nonlinear modeling. To ascertain whether some of the parameters of the comprehensive model were redundant, repetitive optimization was performed with zero model parameters. Specifically, five additional optimization protocols were carried out on the data from all specimens, during which the parameters of each one of the neo-Hookean and four-fiber families were consecutively zeroed and the resulting goodness of fit compared to that when all model parameters were free to vary. Parameter covariance was established by computing the determinant of the correlation matrix **R** for the estimated model parameters, $det\left(R\right)<10^{-4}$ being the limit set to determine over-parameterization.

### 2.5. Statistical Analysis

Individual values, mean values ± standard error (SE), or both are given for our results and calculated parameters. An analysis of variance for repeated measures and a Tukey post-hoc test in SPSS v20.0 (SPSS Inc., Chicago, IL, USA) were used for multiple comparisons among the eight small intestinal segments. Significance was considered at the *p* < 0.05 level.

## 3. Results

### 3.1. Comprehensive Model

**Figure 2:**
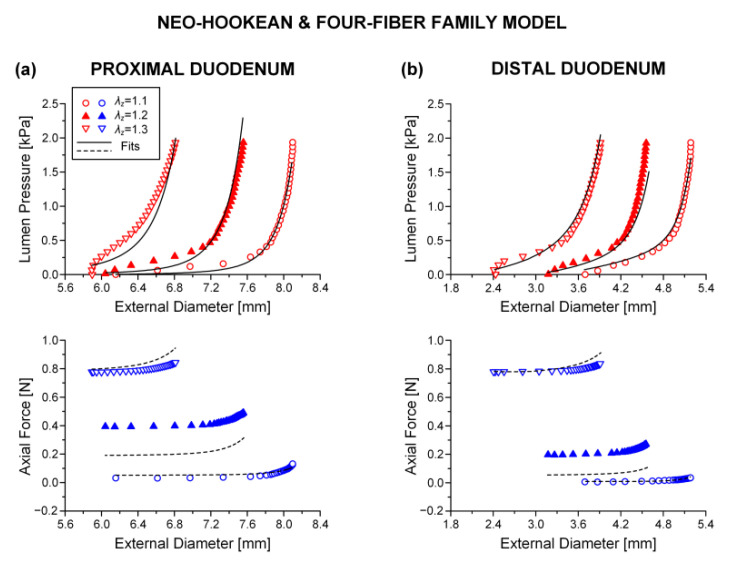
Plot of measured lumen pressure (red color) and axial force (blue color) vs. external diameter data at three different fixed axial stretches $\lambda_{z}=$ {1.1, 1.2, 1.3} for a characteristic specimen from the proximal and distal duodenum and fits (solid lines for lumen pressure and dashed lines for axial force) calculated using the neo-Hookean and four-fiber family microstructure-based model of Equation (1) with the following best-fit model parameters for the (**a**) proximal duodenum: μ= 3.7 × 10^−11^ kPa, $k_{1}^{d}=$ 0.312 kPa, $k_{2}^{d}=$ 16.078, $k_{1}^{a}$ = 8.467 kPa, $k_{2}^{a}$ = 3.143, $k_{1}^{c}$ = 9.5 × 10^−9^ kPa, $k_{2}^{c}$ = 3.922, $a_{0}$ = 0.670 rad, *ε* = 0.321, $R^{2}=$ 0.870, det(R)= 4.7 × 10^−5^ and (**b**) distal duodenum: μ= 5.0 × 10^−6^ kPa, $k_{1}^{d}=$ 0.336 kPa, $k_{2}^{d}=$ 6.940, $k_{1}^{a}$ = 2.470 kPa, $k_{2}^{a}$ = 7.840, $k_{1}^{c}$ = 0.127 kPa, $k_{2}^{c}$ = 3.4 × 10^−8^, $a_{0}$ = 0.308 rad, *ε* = 0.322, $R^{2}=$ 0.881, det(R)= 6.3 × 10^−6^. Data are shown every 0.5 mmHg for clarity.

**Figure 3:**
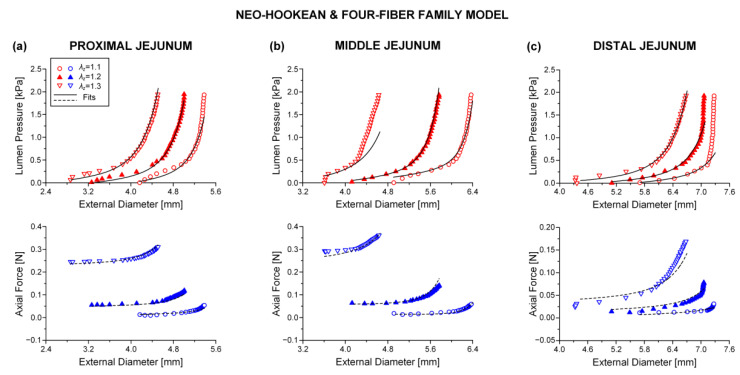
Plot of measured lumen pressure (red color) and axial force (blue color) vs. external diameter data at three different fixed axial stretches $\lambda_{z}=$ {1.1, 1.2, 1.3} for a characteristic specimen from the proximal, middle, and distal jejunum and fits (solid lines for lumen pressure and dashed lines for axial force) calculated using the neo-Hookean and four-fiber family microstructure-based model of Equation (1) with the following best-fit model parameters for the (**a**) proximal jejunum: μ= 7.0 × 10^−12^ kPa, $k_{1}^{d}=$ 3.121 kPa, $k_{2}^{d}=$ 9.743, $k_{1}^{a}$ = 25.331 kPa, $k_{2}^{a}$ = 3.590, $k_{1}^{c}$ = 1.7 × 10^−10^ kPa, $k_{2}^{c}$ = 1.662, $a_{0}$ = 0.691 rad, *ε* = 0.239, $R^{2}=$ 0.927, det(R)= 2.9 × 10^−5^, (**b**) middle jejunum: μ= 1.7 × 10^−13^ kPa, $k_{1}^{d}=$ 0.150 kPa, $k_{2}^{d}=$ 7.627, $k_{1}^{a}$ = 4.723 kPa, $k_{2}^{a}$ = 3.307, $k_{1}^{c}$ = 0.115 kPa, $k_{2}^{c}$ = 3.1 × 10^−7^, $a_{0}$ = 0.310 rad, *ε* = 0.312, $R^{2}=$ 0.871, det(R)= 1.5 × 10^−4^, and (**c**) distal jejunum: μ= 4.7 × 10^−12^ kPa, $k_{1}^{d}=$ 1.237 kPa, $k_{2}^{d}=$ 5.379, $k_{1}^{a}$ = 1.264 kPa, $k_{2}^{a}$ = 0.944, $k_{1}^{c}$ = 2.4 × 10^−10^ kPa, $k_{2}^{c}$ = 0.662, $a_{0}$ = 0.692 rad, *ε* = 0.342, $R^{2}=$ 0.824, det(R)= 7.1 × 10^−4^. Data are shown every 0.5 mmHg for clarity.

**Figure 4:**
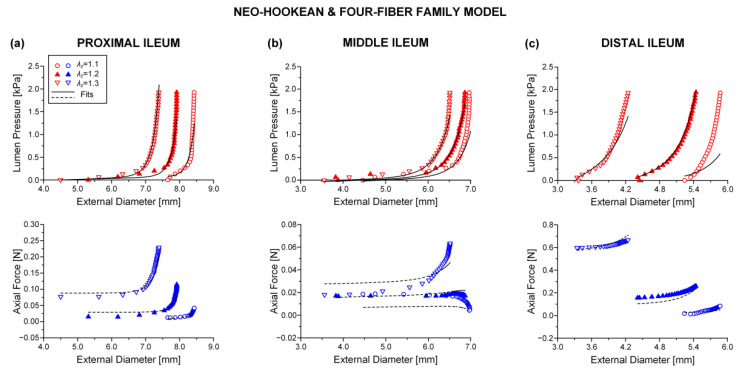
Plot of measured lumen pressure (red color) and axial force (blue color) vs. external diameter data at three different fixed axial stretches $\lambda_{z}=$ {1.1, 1.2, 1.3} for a characteristic specimen from the proximal, middle, and distal ileum and fits (solid lines for lumen pressure and dashed lines for axial force) calculated using the neo-Hookean and four-fiber family microstructure-based model of Equation (1) with the following best-fit model parameters for the (**a**) proximal ileum: μ= 0.103 kPa, $k_{1}^{d}=$ 0.006 kPa, $k_{2}^{d}=$ 6.312, $k_{1}^{a}$ = 1.966 kPa, $k_{2}^{a}$ = 2.102, $k_{1}^{c}$ = 2.4 × 10^−10^ kPa, $k_{2}^{c}$ = 0.008, $a_{0}$ = 0.513 rad, *ε* = 0.260, $R^{2}=$ 0.905, det(R)= 3.5 × 10^−6^, (**b**) middle ileum: μ= 3.2 × 10^−11^ kPa, $k_{1}^{d}=$ 0.694 kPa, $k_{2}^{d}=$ 4.890, $k_{1}^{a}$ = 3.722 kPa, $k_{2}^{a}$ = 7.2 × 10^−11^, $k_{1}^{c}$ = 4.0 × 10^−11^ kPa, $k_{2}^{c}$ = 1.548, $a_{0}$ = 0.852 rad, *ε* = 0.380, $R^{2}=$ 0.788, det(R)= 9.2×10^−6^, and (**c**) distal ileum: μ= 7.0 × 10^−14^ kPa, $k_{1}^{d}=$ 3.629 kPa, $k_{2}^{d}=$ 24.221, $k_{1}^{a}$ = 6.730 kPa, $k_{2}^{a}$ = 4.560, $k_{1}^{c}$ = 0.724 kPa, $k_{2}^{c}$ = 1.023, $a_{0}$ = 0.626 rad, *ε* = 0.333, $R^{2}=$ 0.864, det(R)= 5.3 × 10^−5^. Data are shown every 0.5 mmHg for clarity.

Best-fit parameters of the neo-Hookean and four-fiber family model were found from the pressure-diameter-force data for each small intestinal specimen using the Nelder–Mead algorithm. The nonlinear regression for the parameter estimation reached convergence for all the specimens studied, and the estimated parameters were indeed best fits based on global minimization of *RSS*. Average values of the model parameters for the eight small intestinal segments are listed in Table 1, while individual parameter values are listed in Tables S1–S3 of the Supplementary Materials. Representative examples of fits from each segment are shown in Figure 2, Figure 3 and Figure 4. Good correspondence between the model and data was evidenced on all occasions for the three jejunal segments (Figure 3) and proximal ileum (Figure 4a). Note in Table 1 the high values of the determination coefficient, *R*^2^~0.90, and the low values of root-mean-square error, *ε*~0.26, for these segments. The correspondence between the model and data for the remaining segments was less good (*R*^2^~0.87 and *ε*~0.32), due to the inadequate fit to the force data of the 1.2 axial stretch in five out of the twelve examined duodenal specimens (Figure 2a,b), the inadequate fit to the force data of the 1.1 and 1.3 axial stretches in two out of the six middle ileal specimens (Figure 4b), and the inadequate fit to the pressure data of the 1.1 and/or 1.3 axial stretches in two out of the six distal ileal specimens (Figure 4c).

The values of parameters $\mu ,\;k_{1}^{c},$ and $k_{2}^{c}$ were quite small for all the small intestinal segments in Table 1, reflecting the minor effect of these model terms in characterizing the multiaxial response. The remaining parameters were much greater, and the following inequalities were valid: $k_{1}^{d}<k_{1}^{a}$ and $k_{2}^{d}>k_{2}^{a}$. Material anisotropy was suggested by the $a_{0}$ < 0.785 rad orientation angle of the diagonal-fiber families in all 48 specimens studied except for one ileal specimen. The results of statistical comparisons are catalogued in Table 1, where it is observed that there were pronounced segmental differences. In particular, parameter $k_{1}^{d}$ of the diagonal-fiber families and parameter $k_{1}^{a}$ of the axial-fiber family were significantly (*p* < 0.05) higher in the proximal jejunum than in the majority of segments, and the orientation angle $a_{0}$ was significantly (*p* < 0.05) lower in the distal duodenum than in all other segments. Furthermore, parameters $k_{1}^{c}$ and $k_{2}^{c}$ of the circumferential-fiber family were significantly (*p* < 0.05) higher, in turn, in the middle and distal ileum compared with many other segments. By contrast, there were little segmental differences in parameters μ, $k_{2}^{d}$, and $k_{2}^{a}$ (*p* > 0.05).

A typical caveat of the neo-Hookean and four-fiber family model was that the determinant of correlation matrix det(R) was less than 10^−4^ in most small intestinal specimens (Tables S1–S3 of the Supplementary Materials). On top of that, the error for one or more parameters $\mu ,\;k_{1}^{c},$ and $k_{2}^{c}$ was relatively large as compared to their very small values, and their dependence was almost unity (data not shown), strongly indicating that the comprehensive model was over-parameterized.

### 3.2. Parametric Analysis

**Figure 5:**
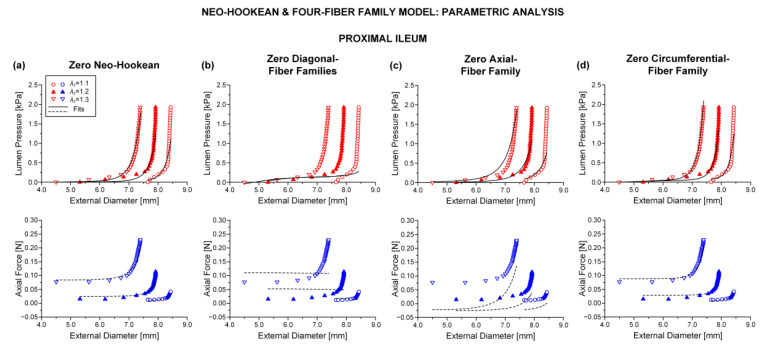
Plots of measured data from the characteristic proximal ileum specimen shown in Figure 4 and fits by the neo-Hookean and four-fiber family model with vanishing (**a**) neo-Hookean term: μ=0 kPa, $k_{1}^{d}=$ 0.027 kPa, $k_{2}^{d}=$ 5.128, $k_{1}^{a}$ = 1.544 kPa, $k_{2}^{a}$ = 2.524, $k_{1}^{c}$ = 8.3 × 10^−11^ kPa, $k_{2}^{c}$ = 0.086, $a_{0}$ = 0.512 rad, *ε* = 0.269, $R^{2}=$ 0.899, det(R)= 1.7 × 10^−5^, (**b**) diagonal-fiber families: μ= 0.307 kPa, $k_{1}^{d}=$ 0 kPa, $k_{2}^{d}=$ 0, = 4.488 kPa, $k_{2}^{a}$ = 0.812, $k_{1}^{c}$ = 0.002 kPa, $k_{2}^{c}$ = 0.295, $a_{0}$ = 0.785 rad, *ε* = 0.687, $R^{2}=$ 0.336, det(R)= 0.001, (**c**) axial-fiber family: μ= 3.5 × 10^−9^ kPa, $k_{1}^{d}=$ 0.110 kPa, $k_{2}^{d}=$ 4.471, $k_{1}^{a}$ = 0 kPa, $k_{2}^{a}$ = 0, $k_{1}^{c}$ = 6.6 × 10^−9^ kPa, $k_{2}^{c}$ = 0.020, $a_{0}$ = 0.487 rad, *ε* = 0.394, $R^{2}=$ 0.782, det(R)= 9.4 × 10^−5^, and (**d**) circumferential-fiber family: μ= 0.102 kPa, $k_{1}^{d}=$ 0.006 kPa, $k_{2}^{d}=$ 6.310, $k_{1}^{a}$ = 1.965 kPa, $k_{2}^{a}$ = 2.102, $k_{1}^{c}$ = 0 kPa, $k_{2}^{c}$ = 0, $a_{0}$ = 0.513 rad, *ε* = 0.260, $R^{2}=$ 0.905, det(R)= 4.8 × 10^−4^.

Shown in Table 2 are the results of successively zeroing each one of the model terms for pooled data from the two duodenal, three jejunal, and three ileal segments; see also Figure 5, illustrating the best fits to characteristic pressure-diameter-force data for the proximal ileum. Upon visual inspection of the graphs and given the ε and $R^{2}$ values in Table 2 resulting from nonlinear regression, it was determined that zeroing the neo-Hookean or circumferential-fiber family terms caused very little change in the goodness of fit; cf. Figure 5a,d with Figure 4a. In stark contrast, note the substantial deterioration of the goodness of fit in the absence of the remaining model terms. The pressure-diameter data were ill-fitted when zeroing the diagonal-fiber family term (Figure 5b), and the force-diameter data were ill-fitted when zeroing the axial-fiber family term (Figure 5c). When both the axial- and circumferential-fiber family terms were zeroed (Table S7 and Figures S1–S3 of the Supplementary Materials), the pressure-diameter data were less accurately fitted in comparison to the neo-Hookean and four-fiber family model, but the force-diameter data were severely underestimated throughout the entire range; cf. Figure S3a with Figure 4a. This neo-Hookean and diagonal-fiber family model was never over-parameterized; refer to the det(R) > 10^−4^ values in Table S7. Still, the computed root-mean-square error and determination coefficient values of ε~0.5 and $R^{2}$~0.5 were indicative of greatly diminished goodness of fit compared to the comprehensive model and the variants without the neo-Hookean or circumferential-fiber family term.

### 3.3. Reduced Model

**Figure 6:**
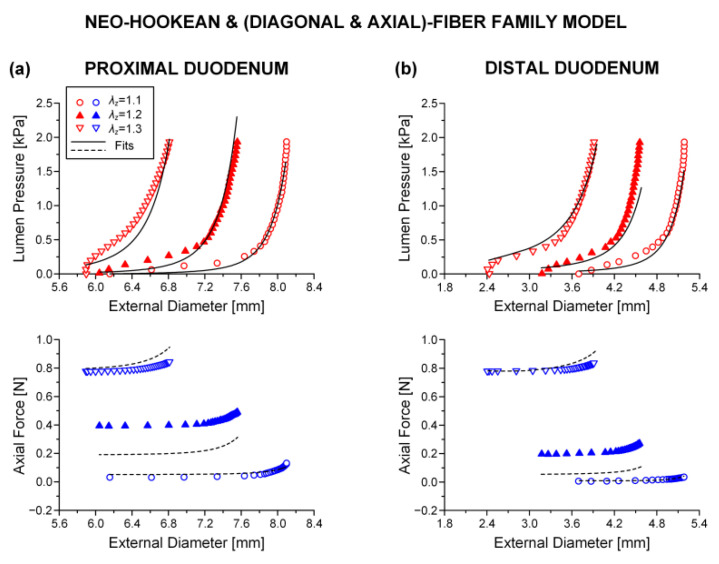
As Figure 2 but using the neo-Hookean and (diagonal and axial)-fiber family model with the following best-fit model parameters for the (**a**) proximal duodenum: μ= 3.7 × 10^−11^ kPa, $k_{1}^{d}=$ 0.312 kPa, $k_{2}^{d}=$ 16.078, $k_{1}^{a}$ = 8.467 kPa, $k_{2}^{a}$ = 3.143, $a_{0}$ = 0.670 rad, *ε* = 0.321, $R^{2}=$ 0.870, det(R)= 0.018 and (**b**) distal duodenum: μ= 3.1 × 10^−6^ kPa, $k_{1}^{d}=$ 0.707 kPa, $k_{2}^{d}=$ 5.332, $k_{1}^{a}$ = 0.915 kPa, $k_{2}^{a}$ = 9.892, $a_{0}$ = 0.319 rad, *ε* = 0.375, $R^{2}=$ 0.838, det(R)= 3.7 × 10^−5^. Data are shown every 0.5 mmHg for clarity.

**Figure 7:**
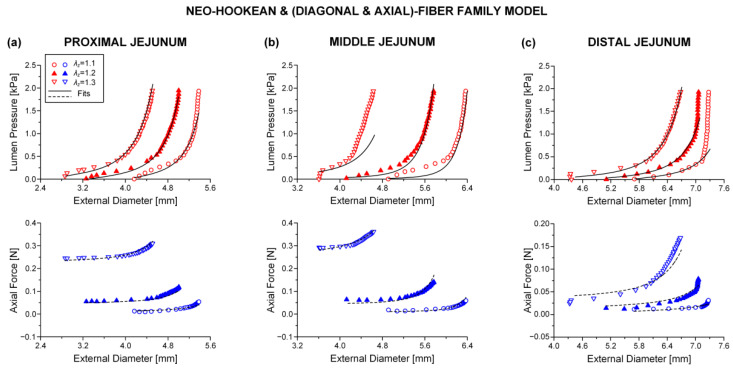
As Figure 3 but using the neo-Hookean and (diagonal and axial)-fiber family model with the following best-fit model parameters for the (**a**) proximal jejunum: μ= 7.0 × 10^−12^ kPa, $k_{1}^{d}=$ 3.121 kPa, $k_{2}^{d}=$ 9.743, $k_{1}^{a}$ = 25.331 kPa, $k_{2}^{a}$ = 3.590, $a_{0}$ = 0.691 rad, *ε* = 0.239, $R^{2}=$ 0.927, det(R)= 0.020, (**b**) middle jejunum: μ= 1.1 × 10^−13^ kPa, $k_{1}^{d}=$ 0.175 kPa, $k_{2}^{d}=$ 7.071, $k_{1}^{a}$ = 2.920 kPa, $k_{2}^{a}$ = 4.464, $a_{0}$ = 0.319 rad, *ε* = 0.353, $R^{2}=$ 0.835, det(R)= 9.6 × 10^−4^, and (**c**) distal jejunum: μ= 4.7 × 10^−12^ kPa, $k_{1}^{d}=$ 1.237 kPa, $k_{2}^{d}=$ 5.379, $k_{1\;}^{a}$ = 1.264 kPa, $k_{2}^{a}$ = 0.944, $a_{0}$ = 0.692 rad, *ε* = 0.342, $R^{2}=$ 0.824, det(R)= 0.011. Data are shown every 0.5 mmHg for clarity.

**Figure 8:**
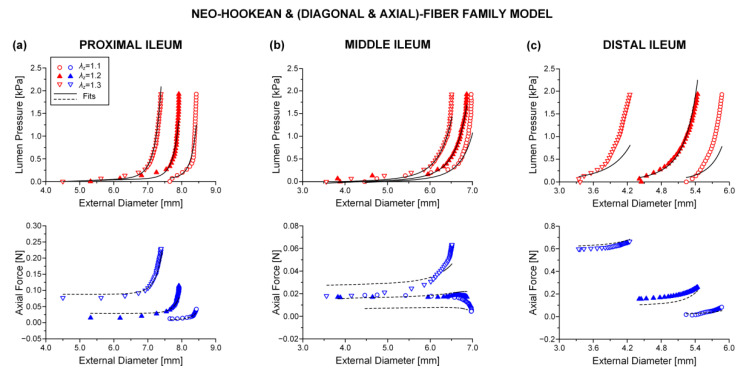
As Figure 4 but using the neo-Hookean and (diagonal and axial)-fiber family model with the following best-fit model parameters for the (**a**) proximal ileum: μ= 0.102 kPa, $k_{1}^{d}=$ 0.006 kPa, $k_{2}^{d}=$ 6.310, $k_{1}^{a}$ = 1.965 kPa, $k_{2}^{a}$ = 2.102, $a_{0}$ = 0.513 rad, *ε* = 0.260, $R^{2}=$ 0.905, det(R)= 4.8 × 10^−4^, (**b**) middle ileum: μ= 3.2 × 10^−11^ kPa, $k_{1}^{d}=$ 0.694 kPa, $k_{2}^{d}=$ 4.890, $k_{1}^{a}$ = 3.722 kPa, $k_{2}^{a}$ = 7.5 × 10^−11^, $a_{0}$ = 0.852 rad, *ε* = 0.380, $R^{2}=$ 0.788, det(R)= 0.002, and (**c**) distal ileum: μ= 6.3 × 10^−14^ kPa, $k_{1}^{d}=$ 3.281 kPa, $k_{2}^{d}=$ 29.283, $k_{1}^{a}$ = 7.588 kPa, $k_{2}^{a}$ = 4.554, $a_{0}$ = 0.678 rad, *ε* = 0.378, $R^{2}=$ 0.825, det(R)= 0.016. Data are shown every 0.5 mmHg for clarity.

The neo-Hookean and (diagonal and axial)-fiber family model was deemed as the preferred reduced model, given that the neo-Hookean term was the sole three-dimensional hyper-elastic body and that in its absence the model would unrealistically predict that no tensile radial loads may be borne by the tissue. The limitation of det(R) < 10^−4^ was avoided, and the ε and $R^{2}$ values of the reduced model resembled those of the comprehensive model; again, being noticeably better for the three jejunal segments and the proximal ileum, in comparison to the ε and $R^{2}$ values found for the proximal and distal duodenum and the middle and distal ileum. See the comparable fitting quality in Figure 2, Figure 3 and Figure 4 and Figure 6, Figure 7 and Figure 8.

Average best-fit parameter values are reported in Table 3, and individual parameter values in Tables S4–S6 (Supplementary Materials). The exact same segmental differences were found as those presented for the comprehensive model, particularly parameter $k_{1}^{d}$ of the diagonal-fiber families and $k_{1}^{a}$ of the axial-fiber family were significantly (*p* < 0.05) increased in the proximal jejunum than in most other segments, and the orientation angle $a_{0}$ was significantly (*p* < 0.05) decreased in the distal duodenum than in all other segments.

### 3.4. Histologic Findings

**Figure 9:**
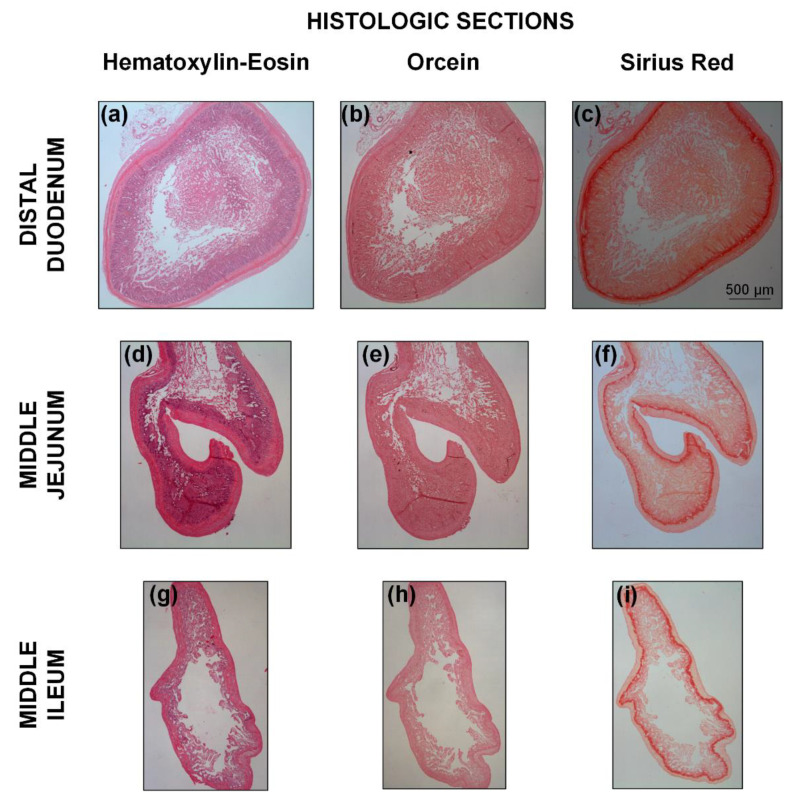
Representative adjacent transverse histologic sections for the (**a**–**c**) distal duodenum, (**d**–**f**) middle jejunum, and (**g**–**i**) middle ileum, stained with hematoxylin-eosin, orcein, and Sirius red for the identification of cells, elastin, and collagen, respectively. The scale bar applies to all the images.

**Figure 10:**
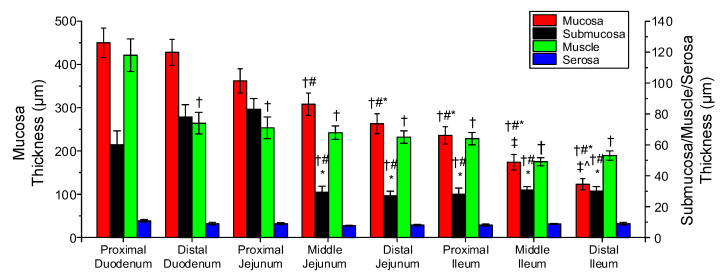
Thickness of the mucosa, submucosa, muscle, and serosa in the small intestinal segments. Symbols †, #, *, ‡, and ^ denote significant difference vs. proximal duodenum, distal duodenum, proximal jejunum, middle jejunum, and distal jejunum.

Figure 9 illustrates the microstructure of different small intestinal segments, and Figure 10 the range of measured mucosa, submucosa, muscle, and serosa thickness along the small intestine. As a general remark, the cellular component was considerable in the muscle layer (Figure 9a,d,g) and less so in the mucosa, but no elastin could be traced altogether, as evidenced by the absence of a dark brown color in the histologic sections stained with orcein (Figure 9b,e,h). Most of the collagen was found in the submucosa and to a lesser degree in the mucosa designated with red color in Sirius red stained sections, and very small amounts were observed in the muscle and serosa (Figure 9c,f,i). The histomorphometric analysis made clear a progressively decreasing mucosa thickness along the small intestine (*p* < 0.05), a significant decrease in submucosa and muscle thickness at the level of the proximal jejunum and distal duodenum, respectively (*p* < 0.05), with minimal change thereafter, and invariant serosa thickness (*p* > 0.05; Figure 10).

## 4. Discussion

### 4.1. General Findings

To the author’s knowledge, this is the first work to implement and evaluate microstructure-based material models specific to the small intestine. Robust parameter values were determined using inflation/extension data covering and exceeding physiologic loadings. Segmental differences in the model parameters were carefully addressed, as characteristic of the differing biomechanical behavior and function with anatomic region. The parametric analysis revealed that the use in the material model of the circumferential-fiber family was unnecessary, suggesting that a reduced neo-Hookean and (axial and diagonal)-fiber family model was preferable and appropriately mimicked the structure of the eight small intestinal segments seen in histologic sections.

### 4.2. Consideration of Microstructure-Based Material Models for the Small Intestine

Our starting microstructure-based model choice was the neo-Hookean and four-fiber family, assigning contributions of circumferentially-, axially-, and diagonally-oriented fiber families to the physiologic and high-pressure macromechanical response, other than the contribution of an isotropic matrix to the low-pressure regime. As evident in soft biologic tissues [13] and particularly in tubular gastrointestinal tissues [2], the recorded inflation/extension data may be divided in three parts: a first part of high extensibility (0–4 mmHg pressures), a second or transitional part (4–12 mmHg) of gradually increasing stiffness incorporating physiologic conditions, and a third part of locked dimensions (> 12 mmHg). Following classic ideas for arterial tissues, the association of elastin and collagen with the deformational response in turn at low and high pressures (stresses) has prompted the consideration of decoupled models with elastin- and collagen-related terms [14]. The neo-Hookean term has been generally believed to reflect the linear and isotropic nature of elastin, while exponential terms have been taken to reflect the nonlinear and anisotropic nature of collagen. The same can be said for collagen with regard to the small intestine, but as the elastin content was very small (according to our histologic staining of rat tissue with orcein; Figure 9b,e,h), it was less likely to play a decisive role. In its place, the passive substance within smooth muscle cells, e.g., the cytoskeleton and cell membrane, may be thought to determine the low-pressure isotropic response of small intestinal tissue, because of their very large content especially in the muscle layer and mixed orientation (Figure 9a,d,g), along with the ground matrix within which the fibrous elements and smooth muscle cells reside; refer to Humphrey [12] for the discussion on the passive contribution of smooth muscle cells in arterial tissues.

When the four- and seven-parameter Fung-type exponential models, alone or together with a quadratic function, were employed by our group, they afforded significantly worse representations to the multiaxial data for the small intestinal wall from eight segments compared to the neo-Hookean and four-fiber family model presently put to test; cf. Table 1, Table 2 and Table 3 in [11] with Table 1 herein. This microstructure-based model was proposed for arterial tissue [15], yet work from our laboratory on similarly structured gastrointestinal tissues—that is, on the large intestine [7] and esophagus [16], as well as on ureteral [17], venous [18], and arterial (elastic/muscular) tissues [19]—have demonstrated its superiority against other phenomenological and microstructure-based constitutive formulations.

An extensive parametric analysis certified that the removal of the circumferential-fiber family from the neo-Hookean and four-fiber family model did not deteriorate the fitting quality to the pressure-diameter-force data of the small intestinal wall. And likewise, the data were equally well captured in the absence of the neo-Hookean term (Table 2), but its presence in the reduced model was necessary, as it was the sole three-dimensional hyper-elastic body. The reduced material model also included diagonal- and axial-fiber families (Table 3). The contributions of the neo-Hookean term and circumferential-fiber family were non-significant, given that quite a few times almost nil values of parameters $\mu ,\;k_{1}^{c},$ and $k_{2}^{c}$ of the full model were found (Tables S1–S3). Another problem of the full model was parameter covariance, as witnessed by the det(R) values, an issue not apparent with the reduced model (cf. Table 1 and Table 3). In the absence from the model of both the axial- and circumferential-fiber families, i.e., for the neo-Hookean and diagonal-fiber family model proposed by Holzapfel et al. [14], the force-diameter data were severely underestimated, and the pressure-diameter data were less accurately represented (Table S7 and Figures S1–S3; Supplementary Materials) in all examined anatomic locations.

### 4.3. Structural Interpretation of Model Parameters: Consideration of Segmental Differences and Physiologic Implications

The rise in circumferential stiffness with load at mid to high pressures was generated by the progressive recruitment and reorientation of the numerous diagonally arranged collagen fibers in the wall, whereas axial stiffness was generated by the axial collagen fibers. This behavior of the small intestine is consistent with the load-bearing mechanisms suggested in [7] for the large intestine, in [16] for the esophagus, and in [17] for ureter, which are histologically comparable tissue types. The near-zero parameters of the neo-Hookean term and circumferential-fiber family, and their minimal contribution to the macro-mechanical response, were likely caused by the small load-bearing capacity of the ground matrix and of muscle in its passive state, and the few collagen fibers with circumferential orientation, so that just minor amounts of stress were born by those components. Consequently, the leftover fiber families dominated the model, with diagonal fibers oriented with respect to the axial direction at an average $a_{0}$ from the eight segments of about 0.614 rad (Table 3). We could not observe lengthier collagen fibers in the axial compared to the circumferential histologic sections, but such a description agrees well with early polarizing optical microscopy, and scanning and transmission electron microscopy studies of rat and bovine intestinal submucosa. Collagen fibers were found to be densely packed in parallel undulating arrays and biaxially oriented at approximately +30° and −30° (i.e., ± 0.523 rad) to the axial direction [20,21,22]. Distinct fiber populations were not discerned with small-angle light scattering, but rather a single population that was centered near the axial direction with wide angular distribution [23].

The layer-specific thickness measurements of the small intestinal wall disclosed obvious segmental variations (Figure 10). Serosa thickness did not vary significantly down the small intestine, but muscle thickness decreased significantly from the proximal to the distal duodenum and little thereafter. Furthermore, mucosa steadily thinned along the organ, and the collagen-rich submucosa was thickest in the proximal jejunum, thinning little toward the duodenal segments and greatly toward the other jejunal and three ileal segments; these measurements substantiated the significant increase of parameter $k_{1}^{d}$ of the diagonal-fiber families and $k_{1}^{a}$ of the axial-fiber family in the proximal jejunum than in the leftover segments (Table 3). Overall, these parameter values and the significant decrease of orientation angle $a_{0}$ of the diagonal-fiber families in the distal duodenum than in all other segments, strongly impacting the biomechanical properties of the small intestinal wall (but unfortunately not histologically corroborated), indicated that the distal duodenum and proximal jejunum were the stiffest of all the segments.

Interestingly, this stiffness distribution is reflective of segmental differences in the physiologic functions of the small intestine. In particular, gastric emptying is an important physiologic event and may be aided by the increased stiffness of the distal duodenum and proximal jejunum. It has been suggested that the duodenum serves as a capacitative resistor [24] and the ileum as a reservoir [25]. The well documented velocity gradient in the small intestine of humans [26] and rats [27] also appears to be related with the segmental differences in the passive biomechanical properties. Investigating those properties in three segments, Storkholm et al. [3] and Dou et al. [5] ascribed the faster transit in the proximal rather than the distal intestine not only to the viscosity of the luminal contents but also to the stiffness gradient throughout the organ, inferring that luminal contents would be slowed to a lesser degree in stiffer segments. The more detailed stiffness distribution documented by this study is in accord with such a proposition, but its physiologic implications remain to be deciphered.

### 4.4. Limitations and Future Studies

Our study had unavoidable limitations that need to be acknowledged. First, the neo-Hookean and four-fiber family and the reduced model provided good representations of the inflation/extension data of the jejunal segments and proximal ileum, but less so of the data of the duodenal segments and the middle and distal ileum (Table 1 and Table 3), which might have been caused by experimental inaccuracies or the need for additional model complexity. Second, although displaying structural analogies, the gross morphology of the mammalian gastrointestinal tract varies markedly among species [28]. Therefore, the documented segmental differences in rats may not be extrapolated to the human condition. Third, understanding the biomechanical response of small intestinal walls from the architectural point of view and validating the selection of a microstructure-based model over another mandates an in-depth knowledge of the individual biomechanical properties of the principal intestinal constituents concerned, namely collagen fibers and muscle cells. The author is unaware of such information in the pertinent literature, but the preparation of selectively digested intestinal tissue with each constituent alone was outside the scope of the present article. It seems reasonable to expect that individual protein and cell properties were similar in the eight segments of the small intestine studied, but this is only a hypothesis at this time. Fourth, no attempt was made to quantify the smooth muscle cell and collagen contents in addition to their orientations in the different wall layers. Correlations with those histologic parameters, except from the qualitative arguments in Section 4.3, would further justify the reported best-fit parameter values. Fifth, even though we examined the small intestinal tissue under supra-physiologic pressures and axial stretches not causing failure, micro-damages may have been caused that cannot be captured by the hyper-elastic model implemented herein. Future studies may attempt to characterize damage phenomena along with the hyper-elastic response, as has been done in [29] for several hyper-elastic models of the literature.

The limitations of our experimental methods were discussed in our previous study [11], but that of considering the small intestinal wall as homogeneous should be re-stated here. Due to the greatly dissimilar composition of the mucosa, submucosa, muscle, and serosa, these layers may be expected to have distinct biomechanical characteristics. Differences in the characteristics of individual mucosal, submucosal, and muscular layers have been shown by our group for the esophagus; refer to [16,30] and the references listed therein. More recently, such differences between the mucosa-submucosa and muscle-serosa have been evidenced for the mouse large intestine and coupled with state-of-the-art determination of the fibrous microstructure with second-harmonic generation confocal microscopy [10,31], again reporting two collagen fiber families oriented at approximately ± 30° to the axial direction for the submucosa. Future studies may endeavor to examine the layer-specific fibrous organization and biomechanical properties of the small intestine, enabling more accurate assessments of transmural stress-strain distributions.

## 5. Conclusions

In spite of the abovementioned drawbacks, our material characterization results emphasized the efficacy of microstructure-based models in representing the multiaxial response of the rat’s small intestine. Our fitting results with the full neo-Hookean and four-fiber family model demonstrated very realistic representations of wide-ranging inflation/extension datasets, but also serious over-parameterization issues related to the presence of the circumferential-fiber family. A reduced neo-Hookean and (axial and diagonal)-fiber family model generated equally good fits without over-parameterization problems, similar to the results of our microstructure-based studies on the large intestine. The current data were also suggestive of segmental variations in layer-specific thickness, reflecting the characterization data along the organ, as anticipated from the physiologic standpoint. In general terms, the distal duodenum and proximal jejunum were the stiffest segments, because of their more axially aligned fibers and thickest collagen-rich submucosa, compared to the remaining segments. The reported variations are of considerable interest, and their physiologic implications merit future attention.

## Figures and Tables

**Table 1:** Parameters of the neo-Hookean and four-fiber family model fitted to experimental data of eight small intestinal segments.

	*μ* [kPa]	$k_{1}^{d}\;[\mathrm{kPa}]$	$k_{2}^{d}\;[-]$	$k_{1}^{a}\;[\mathrm{kPa}]$	$k_{2}^{a}\;[-]$	$k_{1}^{c}\;[\mathrm{kPa}]$	$k_{2}^{c}\;[-]$	$a_{0}\;\;[\mathrm{rad}]$	*ε* [-]	$R^{2}\;\;[-]$	det(R) [-]
PD	0.042 ± 0.042	1.256 ± 0.527 *	17.118 ± 4.047	16.751 ± 6.176	3.788 ± 0.730	0.003 ± 0.003 ^&^	1.546 ± 0.605	0.651 ± 0.029 ^#^	0.325 ± 0.014	0.865 ± 0.011	(14.1 ± 9.2) × 10^−5^
DD	(8.3 ± 8.3) × 10^−7^	0.955 ± 0.216 *	5.964 ± 0.641	10.592 ± 4.936 *	5.235 ± 1.351	0.167 ± 0.031	0.057 ± 0.046 ^@^	0.378 ± 0.028	0.332 ± 0.026	0.857 ± 0.024	(9.0 ± 4.2) × 10^−5^
PJ	(1.8 ± 1.2) × 10^−12^	3.760 ± 0.757	10.521 ± 1.926	49.491 ± 18.385	2.327 ± 0.566	(1.7 ± 1.4) × 10^−9 &^	0.607 ± 0.269 ^@^	0.665 ± 0.028 ^#^	0.287 ± 0.019	0.883 ± 0.018	0.002 ± 0.002
MJ	(2.8 ± 2.7) × 10^−9^	0.615 ± 0.131 *	4.447 ± 1.006	19.571 ± 7.680	3.252 ± 0.988	0.045 ± 0.020	0.205 ± 0.186 ^@^	0.560 ± 0.056 ^#^	0.263 ± 0.015	0.908 ± 0.009	(6.2 ± 2.3) × 10^−5^
DJ	(13.5 ± 8.9) × 10^−11^	1.064 ± 0.191 *	10.974 ± 3.093	16.812 ± 6.229	2.836 ± 0.570	0.090 ± 0.063	1.191 ± 0.649	0.622 ± 0.031 ^#^	0.271 ± 0.019	0.898 ± 0.017	(3.8 ± 1.5) × 10^−4^
PI	0.065 ± 0.048	0.642 ± 0.265 *	9.651 ± 2.983	13.258 ± 4.926 *	3.226 ± 0.420	(3.4 ± 1.8) × 10^−8 &^	0.095 ± 0.092 ^@^	0.599 ± 0.050 ^#^	0.236 ± 0.021	0.919 ± 0.011	(12.0 ± 5.1) × 10^−5^
MI	(2.5 ± 1.7) × 10^−7^	1.261 ± 0.213 *	18.490 ± 5.010	3.713 ± 0.668 *	2.518 ± 0.966	(1.6 ± 1.4) × 10^−4&^	5.298 ± 2.608	0.704 ± 0.048 ^#^	0.319 ± 0.018	0.859 ± 0.019	(5.7 ± 3.9) × 10^−5^
DI	(6.2 ± 3.6) × 10^−9^	2.102 ± 0.658	14.938 ± 2.796	9.541 ± 2.091 *	4.540 ± 0.664	0.240 ± 0.108	1.751 ± 0.283	0.639 ± 0.018 ^#^	0.304 ± 0.020	0.882 ± 0.017	(9.8 ± 4.7) × 10^−5^

PD, DD, PJ, MJ, DJ, PI, MI, and DI denote the proximal duodenum, distal duodenum, proximal jejunum, middle jejunum, distal jejunum, proximal ileum, middle ileum, and distal ileum. *μ*, $k_{1}^{d}$, $k_{2}^{d}$,$\;k_{1}^{a}$, $k_{2}^{a}$, $k_{1}^{c}$, $k_{2}^{c}$, and $a_{0}$ are model parameters, *ε* is the root-mean-square error of fitting, $R^{2}$ is the determination coefficient, and det(R) is the determinant of the correlation matrix for the estimated model parameters. Symbols ^#^, *, ^@^, and ^&^ denote significant difference vs. DD, PJ, MI, and DI. Refer to Tables S1–S3 in the Supplementary Materials for the individual parameter values of the duodenum, jejunum, and ileum, respectively.

**Table 2:** Validation of the parameters of the neo-Hookean and four-fiber family terms for pooled data from the duodenum, jejunum, and ileum.

	*μ* [kPa]	$k_{1}^{d}\;\;[\mathrm{kPa}]$	$k_{2}^{d}\;\;[-]$	$k_{1}^{a}\;\;[\mathrm{kPa}]$	$k_{2}^{a}\;\;[-]$	$k_{1}^{c}\;\;[\mathrm{kPa}]$	$k_{2}^{c}\;\;[-]$	$a_{0}\;\;[\mathrm{rad}]$	*ε* [-]	$R^{2}\;\;[-]$	det(R) [-]
	Zero Neo-Hookean Term										
D	0	1.118 ± 0.330	11.320 ± 2.968	13.155 ± 4.550	4.557 ± 0.920	0.088 ± 0.033	0.857 ± 0.434	0.519 ± 0.051	0.357 ± 0.022	0.835 ± 0.019	(3.4 ± 1.0) × 10^−4^
J	0	1.817 ± 0.480	8.709 ± 1.633	28.980 ± 10.048	2.857 ± 0.491	0.046 ± 0.027	0.738 ± 0.292	0.613 ± 0.029	0.272 ± 0.012	0.895 ± 0.012	(8.4 ± 2.5) × 10^−4^
I	0	1.377 ± 0.319	14.084 ± 3.669	8.338 ± 2.182	3.438 ± 0.522	0.080 ± 0.051	2.542 ± 1.165	0.649 ± 0.030	0.290 ± 0.016	0.883 ± 0.012	(5.6 ± 2.1) × 10^−4^
	Zero Diagonal-Fiber Families										
D	0.103 ± 0.103	0	0	20.853 ± 6.232	3.422 ± 0.701	0.161 ± 0.053	2.483 ± 1.105	0	0.665 ± 0.031	0.442 ± 0.038	0.031 ± 0.011
J	(1.9 ± 1.8) × 10^−9^	0	0	36.421 ± 10.349	2.048 ± 0.347	0.185 ± 0.051	0.964 ± 0.279	0	0.664 ± 0.024	0.392 ± 0.041	0.018 ± 0.004
I	0.064 ± 0.047	0	0	14.093 ± 3.330	2.507 ± 0.400	0.259 ± 0.094	0.890 ± 0.244	0	0.673 ± 0.024	0.389 ± 0.037	0.013 ± 0.003
	Zero Axial-Fiber Family										
D	0.144 ± 0.014	1.939 ± 0.623	10.332 ± 3.270	0	0	0.110 ± 0.054	0.924 ± 0.424	0.487 ± 0.054	0.572 ± 0.041	0.562 ± 0.062	0.010 ± 0.004
J	(1.3 ± 1.2) × 10^−9^	2.529 ± 0.563	7.478 ± 1.339	0	0	0.093 ± 0.065	0.496 ± 0.170	0.572 ± 0.029	0.577 ± 0.032	0.521 ± 0.055	0.011 ± 0.007
I	(2.7 ± 2.7) × 10^−4^	2.979 ± 0.817	9.869 ± 3.525	0	0	0.265 ± 0.164	1.381 ± 0.346	0.562 ± 0.028	0.511 ± 0.023	0.645 ± 0.030	0.002 ± 0.001
	Zero Circumferential-Fiber Family										
D	0.025 ± 0.025	1.185 ± 0.320	11.016 ± 3.043	12.280 ± 4.562	5.127 ± 1.043	0	0	0.528 ± 0.049	0.375 ± 0.022	0.819 ± 0.020	0.007 ± 0.002
J	(1.1 ± 1.1) × 10^−9^	1.804 ± 0.483	8.660 ± 1.630	28.639 ± 10.076	3.075 ± 0.568	0	0	0.619 ± 0.028	0.285 ± 0.014	0.886 ± 0.011	0.015 ± 0.003
I	0.026 ± 0.020	1.298 ± 0.311	14.431 ± 3.755	8.638 ± 2.228	3.329 ± 0.482	0	0	0.671 ± 0.031	0.302 ± 0.019	0.873 ± 0.015	0.008 ± 0.002

D, J, and I denote the duodenum, jejunum, and ileum. *μ*, $k_{1}^{d}$, $k_{2}^{d}$,$\;k_{1}^{a}$, $k_{2}^{a}$, $k_{1}^{c}$, $k_{2}^{c}$, and $a_{0}$ are model parameters, *ε* is the root-mean-square error of fitting, $R^{2}$ is the determination coefficient, and det(R) is the determinant of the correlation matrix for the estimated model parameters.

**Table 3:** Parameters of the neo-Hookean and (diagonal and axial)-fiber family model fitted to experimental data of eight small intestinal segments.

	*μ* [kPa]	$k_{1}^{d}\;\;[\mathrm{kPa}]$	$k_{2}^{d}\;\;[-]$	$k_{1}^{a}\;\;[\mathrm{kPa}]$	$k_{2}^{a}\;\;[-]$	$a_{0}\;\;[\mathrm{rad}]$	*ε* [-]	$R^{2}\;\;[-]$	det(R) [-]
PD	0.042 ± 0.042	1.237 ± 0.517 *	17.196 ± 4.024	16.724 ± 6.175	3.822 ± 0.735	0.652 ± 0.028 ^#^	0.325 ± 0.014	0.865 ± 0.011	0.011 ± 0.002
DD	(5.2 ± 5.2) × 10^−7^	1.127 ± 0.196 *	5.145 ± 0.522	9.183 ± 4.722 *	6.136 ± 1.497	0.397 ± 0.030	0.364 ± 0.024	0.827 ± 0.024	0.004 ± 0.002
PJ	(1.8 ± 1.2) × 10^−12^	3.760 ± 0.757	10.521 ± 1.926	49.491 ± 18.385	2.327 ± 0.566	0.665 ± 0.028 ^#^	0.286 ± 0.019	0.883 ± 0.018	0.028 ± 0.005
MJ	(2.9 ± 2.7) × 10^−9^	0.565 ± 0.130 *	4.549 ± 1.012	19.244 ± 7.821	3.594 ± 1.140	0.570 ± 0.056 ^#^	0.281 ± 0.024	0.894 ± 0.016	0.006 ± 0.002
DJ	(14.5 ± 9.3) × 10^−11^	1.086 ± 0.189 *	10.816 ± 3.110	16.349 ± 6.061	3.030 ± 0.714	0.628 ± 0.029 ^#^	0.284 ± 0.019	0.888 ± 0.017	0.010 ± 0.002
PI	0.065 ± 0.048	0.642 ± 0.265 *	9.650 ± 2.983	13.258 ± 4.927 *	3.226 ± 0.420	0.599 ± 0.050 ^#^	0.236 ± 0.021	0.919 ± 0.011	0.004 ± 0.002
MI	(2.6 ± 1.7) × 10^−7^	1.251 ± 0.214 *	18.474 ± 5.005	3.669 ± 0.671 *	2.520 ± 0.965	0.709 ± 0.045 ^#^	0.323 ± 0.020	0.855 ± 0.021	0.009 ± 0.002
DI	(6.5 ± 3.9) × 10^−9^	1.967 ± 0.648	15.262 ± 3.629	9.918 ± 1.806 *	4.396 ± 0.484	0.695 ± 0.027 ^#^	0.333 ± 0.028	0.858 ± 0.022	0.011 ± 0.003

PD, DD, PJ, MJ, DJ, PI, MI, and DI denote the proximal duodenum, distal duodenum, proximal jejunum, middle jejunum, distal jejunum, proximal ileum, middle ileum, and distal ileum. *μ*, $k_{1}^{d}$, $k_{2}^{d}$,$\;k_{1}^{a}$, $k_{2}^{a}$, and $a_{0}$ are model parameters, *ε* is the root-mean-square error of fitting, $R^{2}$ is the determination coefficient, and det(R) is the determinant of the correlation matrix for the estimated model parameters. Symbols ^#^ and * denote significant difference vs. DD and PJ. Refer to Tables S4–S6 in the Supplementary Materials for the individual parameter values of the duodenum, jejunum, and ileum, respectively.

## Data Availability

The data presented in this study are available on request from the corresponding author. The data are not publicly available due to privacy restrictions.

## Supplementary Materials

The following are available online at https://www.mdpi.com/2306-5354/8/3/32/s1, Figures S1–S3 and Tables S1–S7.
