# Supplementary material for: Variation of Passive Biomechanical Properties of the Small Intestine along Its Length: Microstructure-Based Characterization
Source: Bioengineering (Basel). 2021 Feb 26;8(3):32. doi: 10.3390/bioengineering8030032 (PMC7996941; doi:10.3390/bioengineering8030032)
Supplement: Supplementary file 1 [file bioengineering-08-00032-s001.pdf]

bioengineering

MDPI

Supplementary Material

Variation of Passive Biomechanical Properties of the Small Intestine Along its Length: Microstructure-Based Characterization

Dimitrios P. Sokolis

Laboratory of Biomechanics, Center of Clinical, Experimental Surgery, and Translational Research, Biomedical Research Foundation of the Academy of Athens, 115 27 Athens, Greece; dsokolis@bioacademy.gr; dimitrissokolis@ath.forthnet.gr;

Citation: Sokolis, D.P. Variation of Passive Biomechanical Properties of the Small Intestine Along its Length: Microstructure-Based Characterization. *Bioengineering* **2021**, *8*, 32. https://doi.org/10.3390/bioengineering8030032

Received: 30 November 2020
Accepted: 19 February 2021
Published: 26 February 2021

Publisher's Note: MDPI stays neutral with regard to jurisdictional claims in published maps and institutional affiliations.

Copyright: © 2021 by the author. Licensee MDPI, Basel, Switzerland. This article is an open access article distributed under the terms and conditions of the Creative Commons Attribution (CC BY) license (http://creativecommons.org/licenses/by/4.0/).

Table S1. Parameters of the neo-Hookean and four-fiber family model fitted to experimental data of two duodenal segments.

	μ [kPa]	k_1^d [kPa]	k_2^d [-]	k_1^a [kPa]	k_2^a [-]	k_1^c [kPa]	k_2^c [-]	a_0 [rad]	ε [-]	R^2[-]	$det(\mathbf{R})$ [-]
Proximal Duodenum											
Rat 1	1.8×10^{-12}	2.689	11.016	3.017	5.760	0.021	2.770	0.522	0.338	0.869	1.9×10^{-4}
Rat 2	3.7×10^{-11}	0.312	16.078	8.467	3.143	9.5×10^{-9}	3.922	0.670	0.321	0.870	4.7×10^{-5}
Rat 3	1.6×10^{-12}	2.997	11.739	3.181	6.052	6.3×10^{-9}	0.824	0.727	0.374	0.830	5.8×10^{-4}
Rat 4	0.255	0.292	9.055	42.234	1.562	3.1×10^{-7}	0.003	0.676	0.297	0.878	1.4×10^{-5}
Rat 5	1.7×10^{-12}	0.062	35.990	20.716	2.521	1.8×10^{-8}	0.718	0.679	0.344	0.838	6.8×10^{-6}
Rat 6	2.3×10^{-12}	1.185	18.832	22.893	3.692	5.7×10^{-7}	1.039	0.634	0.276	0.903	7.3×10^{-6}
Mean	0.042	1.256	17.118	16.751	3.788	0.003	1.546	0.651	0.325	0.865	(14.1 ± 9.2)
±SE	± 0.042	± 0.527*	± 4.047	± 6.176	± 0.730	± 0.003&	± 0.605	± 0.029#	± 0.014	± 0.011	$\times 10^{-5}$
Distal Duodenum											
Rat 1	5.1×10^{-13}	0.739	7.695	3.095	4.163	0.123	0.049	0.321	0.224	0.932	5.9×10^{-5}
Rat 2	1.8×10^{-10}	1.083	7.253	0.562	10.383	0.288	0.282	0.496	0.357	0.856	2.8×10^{-5}
Rat 3	5.0×10^{-6}	0.336	6.940	2.470	7.840	0.127	3.4×10^{-8}	0.308	0.322	0.881	6.3×10^{-6}
Rat 4	2.6×10^{-10}	0.655	4.338	15.615	1.838	0.230	3.3×10^{-5}	0.397	0.402	0.777	2.4×10^{-4}
Rat 5	6.3×10^{-14}	1.881	3.978	32.530	2.291	0.091	1.9×10^{-8}	0.387	0.382	0.800	2.0×10^{-4}
Rat 6	7.5×10^{-10}	1.038	5.581	9.279	4.892	0.145	0.008	0.356	0.307	0.893	7.9×10^{-6}
Mean	(8.3 ± 8.3)	0.955	5.964	10.592	5.235	0.167	0.057	0.378	0.332	0.857	(9.0 ± 4.2)
±SE	$\times 10^{-7}$	± 0.216*	± 0.641	± 4.936*	± 1.351	± 0.031	± 0.046@	± 0.028	± 0.026	± 0.024	$\times 10^{-5}$

μ, k_1^d, k_2^d, k_1^a, k_2^a, k_1^c, k_2^c, and a_0 are model parameters, ε is the root-mean-square error of fitting, R^2 is the determination coefficient, and $det(\mathbf{R})$ is the determinant of the correlation matrix for the estimated model parameters. Symbols #, *, @, and & denote significant difference vs. distal duodenum, proximal jejunum, middle ileum, and distal ileum.

Table S2. Parameters of the neo-Hookean and four-fiber family model fitted to experimental data of three jejunal segments.

	μ [kPa]	k_1^d [kPa]	k_2^d [-]	k_1^a [kPa]	k_2^a [-]	k_1^c [kPa]	k_2^c [-]	a_0 [rad]	ε [-]	R^2[-]	$det(\mathbf{R})$ [-]
Proximal Jejunum											
Rat 1	2.3×10^{-18}	2.914	7.978	131.866	5.1×10^{-8}	8.8×10^{-9}	1.016	0.630	0.335	0.821	7.3×10^{-4}
Rat 2	7.0×10^{-12}	3.121	9.743	25.331	3.590	1.7×10^{-10}	1.662	0.691	0.239	0.927	2.9×10^{-5}
Rat 3	1.6×10^{-13}	2.527	4.539	19.983	3.372	1.2×10^{-9}	0.200	0.553	0.221	0.935	1.1×10^{-4}
Rat 4	1.3×10^{-15}	7.472	12.307	61.371	1.398	4.2×10^{-11}	0.028	0.736	0.334	0.843	0.002
Rat 5	4.7×10^{-18}	2.894	18.579	7.567	3.116	1.6×10^{-13}	8.5×10^{-5}	0.723	0.297	0.882	0.012
Rat 6	3.8×10^{-12}	3.632	9.983	50.829	2.483	2.3×10^{-10}	0.734	0.654	0.293	0.891	3.6×10^{-5}
Mean	(1.8 ± 1.2)	3.760	10.521	49.491	2.327	(1.7 ± 1.4)	0.607	0.665	0.287	0.883	0.002
±SE	$\times 10^{-12}$	± 0.757	± 1.926	± 18.385	± 0.566	$\times 10^{-9}$ [&]	± 0.269[@]	± 0.028[#]	± 0.019	± 0.018	± 0.002
Middle Jejunum											
Rat 1	2.4×10^{-10}	0.688	6.816	1.135	7.619	0.083	1.133	0.568	0.279	0.907	1×10^{-5}
Rat 2	1.7×10^{-8}	0.765	1.951	26.981	1.768	1.4×10^{-6}	0.002	0.732	0.264	0.904	7.1×10^{-6}
Rat 3	1.7×10^{-13}	0.150	7.627	4.723	3.307	0.115	3.1×10^{-7}	0.310	0.312	0.871	1.5×10^{-4}
Rat 4	1.7×10^{-10}	0.312	1.572	53.056	0.439	2.5×10^{-9}	1.1×10^{-4}	0.562	0.223	0.925	3.4×10^{-5}
Rat 5	1.4×10^{-11}	1.014	4.621	17.141	3.432	0.067	9.7×10^{-8}	0.597	0.218	0.937	9.5×10^{-5}
Rat 6	4.9×10^{-11}	0.764	4.097	14.392	2.949	0.006	0.096	0.592	0.284	0.902	7.3×10^{-5}
Mean	(2.8 ± 2.7)	0.615	4.447	19.571	3.252	0.045	0.205	0.560	0.263	0.908	(6.2 ± 2.3)
±SE	$\times 10^{-9}$	± 0.131[*]	± 1.006	± 7.680	± 0.988	± 0.020	± 0.186[@]	± 0.056[#]	± 0.015	± 0.009	$\times 10^{-5}$
Distal Jejunum											
Rat 1	4.7×10^{-12}	1.237	5.379	1.264	0.944	2.4×10^{-10}	0.662	0.692	0.342	0.824	7.1×10^{-4}
Rat 2	2.6×10^{-10}	1.013	10.308	1.515	5.135	0.033	4.332	0.653	0.234	0.929	4.3×10^{-5}
Rat 3	4.5×10^{-16}	1.845	25.776	12.598	2.710	6.0×10^{-10}	1.000	0.653	0.279	0.896	7.7×10^{-4}
Rat 4	1.1×10^{-15}	0.881	6.522	35.455	2.571	3.3×10^{-9}	0.196	0.619	0.209	0.942	6.5×10^{-4}
Rat 5	1.4×10^{-11}	0.423	6.925	34.720	3.457	0.392	3.3×10^{-7}	0.476	0.293	0.889	1.3×10^{-5}
Rat 6	5.3×10^{-10}	0.982	10.932	15.321	2.199	0.116	0.958	0.638	0.266	0.909	6.4×10^{-5}
Mean	(13.5 ± 8.9)	1.064	10.974	16.812	2.836	0.090	1.191	0.622	0.271	0.898	(3.8 ± 1.5)
±SE	$\times 10^{-11}$	± 0.191[*]	± 3.093	± 6.229	± 0.570	± 0.063	± 0.649	± 0.031[#]	± 0.019	± 0.017	$\times 10^{-4}$

μ, k_1^d, k_2^d, k_1^a, k_2^a, k_1^c, k_2^c, and a_0 are model parameters, ε is the root-mean-square error of fitting, R^2 is the determination coefficient, and $det(\mathbf{R})$ is the determinant of the correlation matrix for the estimated model parameters. Symbols [#], [*], [@], and [&] denote significant difference vs. distal duodenum, proximal jejunum, middle ileum, and distal ileum.

Table S3. Parameters of the neo-Hookean and four-fiber family model fitted to experimental data of three ileal segments.

	μ [kPa]	k_1^d [kPa]	k_2^d [-]	k_1^a [kPa]	k_2^a [-]	k_1^c [kPa]	k_2^c [-]	a_0 [rad]	ε [-]	R^2[-]	$det(\mathbf{R})$ [-]
Proximal Ileum											
Rat 1	0.103	0.006	6.312	1.966	2.102	2.4×10^{-10}	0.008	0.513	0.260	0.905	3.5×10^{-6}
Rat 2	9.4×10^{-8}	0.368	5.973	2.629	3.167	5.3×10^{-8}	6.4×10^{-5}	0.713	0.293	0.895	1.2×10^{-5}
Rat 3	1.2×10^{-12}	1.896	24.494	6.638	2.388	4.2×10^{-10}	0.557	0.774	0.250	0.913	2.0×10^{-4}
Rat 4	0.289	0.484	6.933	31.323	5.009	2.1×10^{-9}	1.9×10^{-7}	0.485	0.143	0.970	3.0×10^{-4}
Rat 5	1.4×10^{-11}	0.498	6.229	24.089	3.111	1.1×10^{-7}	0.007	0.501	0.247	0.919	1.8×10^{-4}
Rat 6	3.6×10^{-8}	0.597	7.963	12.902	3.582	3.6×10^{-8}	4.6×10^{-5}	0.605	0.223	0.912	2.4×10^{-5}
Mean	0.065	0.642	9.651	13.258	3.226	(3.4 ± 1.8)	0.095	0.599	0.236	0.919	(12.0 ± 5.1)
±SE	± 0.048	± 0.265*	± 2.983	± 4.926*	± 0.420	$\times 10^{-8}$&	± 0.092@	± 0.050#	± 0.021	± 0.011	$\times 10^{-5}$
Middle Ileum											
Rat 1	2.9×10^{-15}	0.973	22.692	2.798	8.2×10^{-9}	1.0×10^{-4}	1.268	0.526	0.331	0.827	5.1×10^{-6}
Rat 2	7.3×10^{-14}	1.670	20.334	1.865	6.285	8.8×10^{-4}	7.704	0.639	0.357	0.846	3.6×10^{-5}
Rat 3	9.5×10^{-7}	2.101	4.820	3.354	2.570	1.5×10^{-6}	17.322	0.805	0.282	0.895	5.9×10^{-6}
Rat 4	6.5×10^{-11}	1.013	37.314	6.710	3.562	7.9×10^{-8}	1.206	0.727	0.264	0.905	2.5×10^{-4}
Rat 5	3.2×10^{-11}	0.694	4.890	3.722	7.2×10^{-11}	4.0×10^{-11}	1.548	0.852	0.380	0.788	9.2×10^{-6}
Rat 6	5.7×10^{-7}	1.119	20.892	3.831	2.692	6.2×10^{-6}	2.736	0.673	0.301	0.893	3.4×10^{-5}
Mean	(2.5 ± 1.7)	1.261	18.490	3.713	2.518	(1.6 ± 1.4)	5.298	0.704	0.319	0.859	(5.7 ± 3.9)
±SE	$\times 10^{-7}$	± 0.213*	± 5.010	± 0.668*	± 0.966	$\times 10^{-4}$&	± 2.608	± 0.048#	± 0.018	± 0.019	$\times 10^{-5}$
Distal Ileum											
Rat 1	1.1×10^{-8}	0.490	8.284	14.127	4.060	1.8×10^{-12}	1.563	0.617	0.263	0.911	3.3×10^{-5}
Rat 2	2.2×10^{-8}	0.507	19.163	6.840	3.934	0.261	2.972	0.650	0.389	0.806	1.7×10^{-4}
Rat 3	3.6×10^{-11}	4.391	16.072	12.219	2.642	2.1×10^{-9}	1.301	0.713	0.254	0.914	3.0×10^{-4}
Rat 4	7.0×10^{-14}	3.629	24.221	6.730	4.560	0.724	1.023	0.626	0.333	0.864	5.3×10^{-5}
Rat 5	1.1×10^{-12}	1.584	5.791	2.101	7.544	0.209	1.565	0.582	0.290	0.900	3.9×10^{-6}
Rat 6	4.4×10^{-9}	2.008	16.098	15.231	4.501	0.246	2.084	0.645	0.297	0.894	2.6×10^{-5}
Mean	(6.2 ± 3.6)	2.102	14.938	9.541	4.540	0.240	1.751	0.639	0.304	0.882	(9.8 ± 4.7)
±SE	$\times 10^{-9}$	± 0.658	± 2.796	± 2.091*	± 0.664	± 0.108	± 0.283	± 0.018#	± 0.020	± 0.017	$\times 10^{-5}$

μ, k_1^d, k_2^d, k_1^a, k_2^a, k_1^c, k_2^c, and a_0 are model parameters, ε is the root-mean-square error of fitting, R^2 is the determination coefficient, and $det(\mathbf{R})$ is the determinant of the correlation matrix for the estimated model parameters. Symbols #, *, @, and & denote significant difference vs. distal duodenum, proximal jejunum, middle ileum, and distal ileum.

Table S4. Parameters of the neo-Hookean and (diagonal and axial)-fiber family model fitted to experimental data of two duodenal segments.

	μ [kPa]	k_1^d [kPa]	k_2^d [-]	k_1^a [kPa]	k_2^a [-]	a_0 [rad]	ε [-]	R^2[-]	$det(\mathbf{R})$ [-]
Proximal Duodenum									
Rat 1	1.9×10^{-12}	2.575	11.484	2.979	5.813	0.527	0.339	0.868	0.011
Rat 2	3.7×10^{-11}	0.312	16.078	8.467	3.143	0.670	0.321	0.870	0.018
Rat 3	1.6×10^{-12}	2.997	11.739	3.181	6.052	0.727	0.374	0.831	0.016
Rat 4	0.255	0.292	9.055	42.234	1.562	0.676	0.297	0.878	0.007
Rat 5	1.8×10^{-12}	0.062	35.990	20.716	2.521	0.679	0.344	0.838	0.004
Rat 6	2.3×10^{-12}	1.185	18.832	22.768	3.839	0.634	0.277	0.903	0.009
Mean	0.042	1.237	17.196	16.724	3.822	0.652	0.325	0.865	0.011
±SE	± 0.042	± 0.517*	± 4.024	± 6.175	± 0.735	± 0.028#	± 0.014	± 0.011	± 0.002
Distal Duodenum									
Rat 1	5.7×10^{-13}	1.070	6.474	1.178	6.433	0.337	0.281	0.894	0.002
Rat 2	2.2×10^{-10}	1.056	6.613	0.445	10.976	0.522	0.394	0.807	2.2×10^{-4}
Rat 3	3.1×10^{-6}	0.707	5.332	0.915	9.892	0.319	0.375	0.838	3.7×10^{-5}
Rat 4	3.5×10^{-10}	0.749	3.643	12.289	2.426	0.430	0.439	0.735	0.003
Rat 5	6.3×10^{-14}	2.032	3.750	30.399	2.447	0.395	0.384	0.798	0.013
Rat 6	7.4×10^{-10}	1.145	5.057	9.871	4.638	0.378	0.311	0.887	0.008
Mean	(5.2 ± 5.2)	1.127	5.145	9.183	6.136	0.397	0.364	0.827	0.004
±SE	$\times 10^{-7}$	± 0.196*	± 0.522	± 4.722*	± 1.497	± 0.030	± 0.024	± 0.024	± 0.002

μ, k_1^d, k_2^d, k_1^a, k_2^a, k_1^c, k_2^c, and a_0 are model parameters, ε is the root-mean-square error of fitting, R^2 is the determination coefficient, and $det(\mathbf{R})$ is the determinant of the correlation matrix for the estimated model parameters. Symbols # and * denote significant difference vs. distal duodenum and proximal jejunum.

Table S5. Parameters of the neo-Hookean and (diagonal and axial)-fiber family model fitted to experimental data of three jejunal segments.

	μ [kPa]	k_1^d [kPa]	k_2^d [-]	k_1^a [kPa]	k_2^a [-]	a_0 [rad]	ε [-]	R^2[-]	$det(\mathbf{R})$ [-]
Proximal Jejunum									
Rat 1	2.3×10^{-18}	2.914	7.978	131.866	5.1×10^{-8}	0.630	0.335	0.821	0.043
Rat 2	7.0×10^{-12}	3.121	9.743	25.331	3.590	0.691	0.239	0.927	0.020
Rat 3	1.6×10^{-13}	2.527	4.539	19.983	3.372	0.553	0.221	0.935	0.017
Rat 4	1.3×10^{-15}	7.472	12.307	61.371	1.398	0.736	0.334	0.843	0.044
Rat 5	4.7×10^{-18}	2.894	18.579	7.567	3.116	0.723	0.296	0.882	0.021
Rat 6	3.8×10^{-12}	3.632	9.983	50.829	2.483	0.654	0.293	0.891	0.024
Mean	(1.8 ± 1.2)	3.760	10.521	49.491	2.327	0.665	0.286	0.883	0.028
±SE	$\times 10^{-12}$	± 0.757	± 1.926	± 18.385	± 0.566	± 0.028#	± 0.019	± 0.018	± 0.005
Middle Jejunum									
Rat 1	3.9×10^{-10}	0.377	7.500	0.800	8.538	0.608	0.346	0.858	9.5×10^{-4}
Rat 2	1.7×10^{-8}	0.765	1.951	26.981	1.768	0.732	0.264	0.904	0.010
Rat 3	1.1×10^{-13}	0.175	7.071	2.920	4.464	0.319	0.353	0.835	9.6×10^{-4}
Rat 4	1.7×10^{-10}	0.312	1.572	53.056	0.439	0.562	0.223	0.925	0.009
Rat 5	1.5×10^{-11}	0.973	4.698	16.932	3.459	0.600	0.220	0.936	0.010
Rat 6	4.8×10^{-11}	0.786	4.503	14.775	2.896	0.597	0.280	0.905	0.008
Mean	(2.9 ± 2.7)	0.565	4.549	19.244	3.594	0.570	0.281	0.894	0.006
±SE	$\times 10^{-9}$	± 0.130*	± 1.012	± 7.821	± 1.140	± 0.056#	± 0.024	± 0.016	± 0.002
Distal Jejunum									
Rat 1	4.7×10^{-12}	1.237	5.379	1.264	0.944	0.692	0.342	0.824	0.011
Rat 2	3.2×10^{-10}	1.134	9.560	1.009	6.128	0.674	0.281	0.898	0.003
Rat 3	4.5×10^{-16}	1.845	25.776	12.598	2.710	0.653	0.279	0.896	0.013
Rat 4	1.1×10^{-15}	0.881	6.522	35.455	2.571	0.619	0.209	0.942	0.017
Rat 5	1.4×10^{-11}	0.437	6.729	32.446	3.630	0.492	0.328	0.861	0.007
Rat 6	5.3×10^{-10}	0.982	10.932	15.321	2.198	0.638	0.266	0.909	0.009
Mean	(14.5 ± 9.3)	1.086	10.816	16.349	3.030	0.628	0.284	0.888	0.010
±SE	$\times 10^{-11}$	± 0.189*	± 3.110	± 6.061	± 0.714	± 0.029#	± 0.019	± 0.017	± 0.002

μ, k_1^d, k_2^d, k_1^a, k_2^a, k_1^c, k_2^c, and a_0 are model parameters, ε is the root-mean-square error of fitting, R^2 is the determination coefficient, and $det(\mathbf{R})$ is the determinant of the correlation matrix for the estimated model parameters. Symbols # and * denote significant difference vs. distal duodenum and proximal jejunum.

Table S6. Parameters of the neo-Hookean and (diagonal and axial)-fiber family model fitted to experimental data of three ileal segments.

	μ [kPa]	k_1^d [kPa]	k_2^d [-]	k_1^a [kPa]	k_2^a [-]	a_0 [rad]	ε [-]	R^2[-]	$det(\mathbf{R})$ [-]
Proximal Ileum									
Rat 1	0.102	0.006	6.310	1.965	2.102	0.513	0.260	0.905	4.8×10^{-4}
Rat 2	9.4×10^{-8}	0.368	5.973	2.629	3.167	0.713	0.293	0.895	0.003
Rat 3	1.2×10^{-12}	1.896	24.494	6.638	2.388	0.774	0.250	0.913	0.011
Rat 4	0.289	0.484	6.933	31.323	5.009	0.485	0.143	0.970	6.6×10^{-4}
Rat 5	1.4×10^{-11}	0.498	6.229	24.089	3.111	0.501	0.247	0.919	0.007
Rat 6	3.5×10^{-8}	0.597	7.963	12.902	3.582	0.605	0.222	0.913	0.004
Mean	0.065	0.642	9.650	13.258	3.226	0.599	0.236	0.919	0.004
±SE	± 0.048	± 0.265*	± 2.983	± 4.927*	± 0.420	± 0.050#	± 0.021	± 0.011	± 0.002
Middle Ileum									
Rat 1	2.9×10^{-15}	0.922	22.515	2.711	8.7×10^{-9}	0.554	0.358	0.799	0.013
Rat 2	7.5×10^{-14}	1.659	20.402	1.873	6.280	0.639	0.357	0.847	0.007
Rat 3	9.8×10^{-7}	2.098	4.833	3.347	2.587	0.808	0.282	0.895	0.007
Rat 4	6.5×10^{-11}	1.013	37.314	6.710	3.562	0.727	0.264	0.906	0.017
Rat 5	3.2×10^{-11}	0.694	4.890	3.722	7.5×10^{-11}	0.852	0.380	0.788	0.002
Rat 6	5.6×10^{-7}	1.119	20.892	3.831	2.694	0.673	0.299	0.896	0.006
Mean	(2.6 ± 1.7)	1.251	18.474	3.669	2.520	0.709	0.323	0.855	0.009
±SE	$\times 10^{-7}$	± 0.214*	± 5.005	± 0.671*	± 0.965	± 0.045#	± 0.020	± 0.021	± 0.002
Distal Ileum									
Rat 1	1.1×10^{-8}	0.490	8.284	14.127	4.060	0.617	0.263	0.912	0.008
Rat 2	2.4×10^{-8}	0.487	19.225	6.291	4.165	0.669	0.407	0.787	0.010
Rat 3	3.6×10^{-11}	4.391	16.072	12.219	2.642	0.713	0.254	0.914	0.021
Rat 4	6.3×10^{-14}	3.281	29.283	7.588	4.554	0.678	0.378	0.825	0.016
Rat 5	1.5×10^{-13}	1.189	3.711	4.344	6.316	0.816	0.394	0.816	9.7×10^{-4}
Rat 6	4.4×10^{-9}	1.963	14.996	14.937	4.638	0.678	0.302	0.893	0.009
Mean	(6.5 ± 3.9)	1.967	15.262	9.918	4.396	0.695	0.333	0.858	0.011
±SE	$\times 10^{-9}$	± 0.648	± 3.629	± 1.806*	± 0.484	± 0.027#	± 0.028	± 0.022	± 0.003

μ, k_1^d, k_2^d, k_1^a, k_2^a, k_1^c, k_2^c, and a_0 are model parameters, ε is the root-mean-square error of fitting, R^2 is the determination coefficient, and $det(\mathbf{R})$ is the determinant of the correlation matrix for the estimated model parameters. Symbols # and * denote significant difference vs. distal duodenum and proximal jejunum.

Table S7. Parameters of the neo-Hookean and diagonal-fiber family model fitted to experimental data of eight small intestinal segments.

	μ [kPa]	k_1^d [kPa]	k_2^d [-]	a_0 [rad]	ε [-]	R^2[-]	$det(\mathbf{R})$ [-]
Duodenum							
Proximal	0.029 ± 0.029	1.803 ± 0.957	16.132 ± 5.656	0.641 ± 0.034	0.628 ± 0.045	0.483 ± 0.084	0.052 ± 0.019
Distal	(1.2 ± 1.2) × 10^{-8}	4.824 ± 3.896	5.337 ± 0.995	0.370 ± 0.037	0.530 ± 0.051	0.635 ± 0.066	0.039 ± 0.008
Jejunum							
Proximal	(1.3 ± 1.3) × 10^{-12}	4.887 ± 0.859	8.779 ± 1.709	0.643 ± 0.035	0.627 ± 0.048	0.429 ± 0.107	0.101 ± 0.012
Middle	(3.8 ± 3.8) × 10^{-9}	0.641 ± 0.139	4.965 ± 1.234	0.522 ± 0.060	0.596 ± 0.045	0.506 ± 0.086	0.035 ± 0.008
Distal	(3.7 ± 2.8) × 10^{-12}	1.527 ± 0.391	9.856 ± 3.246	0.598 ± 0.029	0.536 ± 0.057	0.600 ± 0.074	0.056 ± 0.014
Ileum							
Proximal	(2.2 ± 2.1) × 10^{-8}	2.547 ± 1.836	4.927 ± 0.869	0.517 ± 0.026	0.505 ± 0.034	0.647 ± 0.047	0.053 ± 0.036
Middle	0.220 ± 0.220	2.593 ± 0.657	17.916 ± 9.882	0.601 ± 0.052	0.495 ± 0.038	0.665 ± 0.043	0.098 ± 0.021
Distal	(4.5 ± 4.4) × 10^{-9}	2.422 ± 0.926	15.020 ± 4.623	0.682 ± 0.026	0.634 ± 0.016	0.492 ± 0.024	0.054 ± 0.009

μ, k_1^d, k_2^d, and a_0 are model parameters, ε is the root-mean-square error of fitting, R^2 is the determination coefficient, and $det(\mathbf{R})$ is the determinant of correlation matrix for the estimated model parameters.

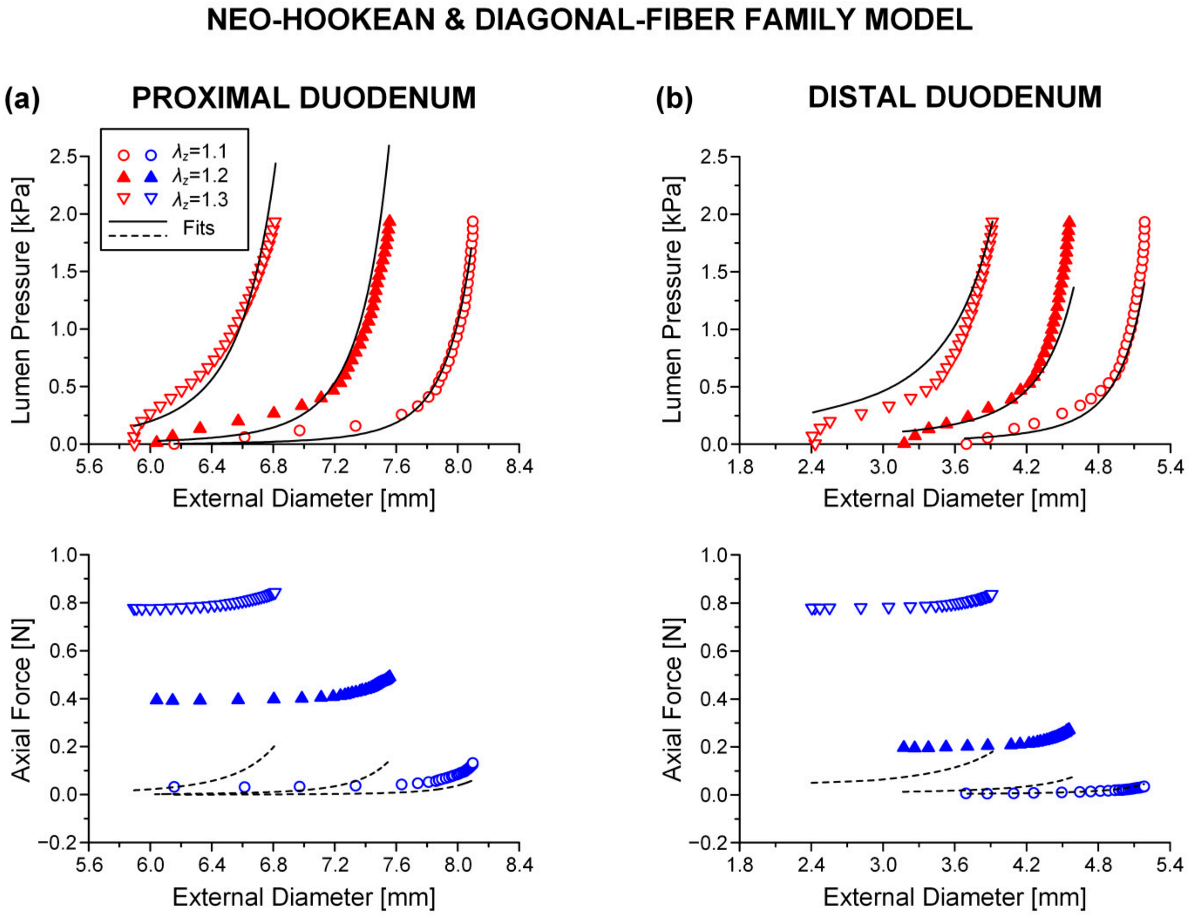

Figure S1. Plot of measured lumen pressure (red color) and axial force (blue color) vs. external diameter data at three different fixed axial stretches λ_z ={1.1, 1.2, 1.3} for a characteristic specimen from the proximal and distal duodenum and fits (solid lines for lumen pressure and dashed lines for axial force) calculated using the neo-Hookean and diagonal-fiber family microstructure-based model with the following best-fit model parameters for the (**a**) proximal duodenum: $\mu = 3.9 \times 10^{-11}$ kPa, $k_1^d = 0.327$ kPa, $k_2^d = 16.493$, $\alpha_0 = 0.663$ rad, $\varepsilon = 0.626$, $R^2 = 0.507$, $det(\mathbf{R}) = 0.033$ and (**b**) distal duodenum: $\mu = 5.8 \times 10^{-8}$ kPa, $k_1^d = 0.908$ kPa, $k_2^d = 5.215$, $\alpha_0 = 0.314$ rad, $\varepsilon = 0.518$, $R^2 = 0.691$, $det(\mathbf{R}) = 0.028$. Data are shown every 0.5 mmHg for clarity.

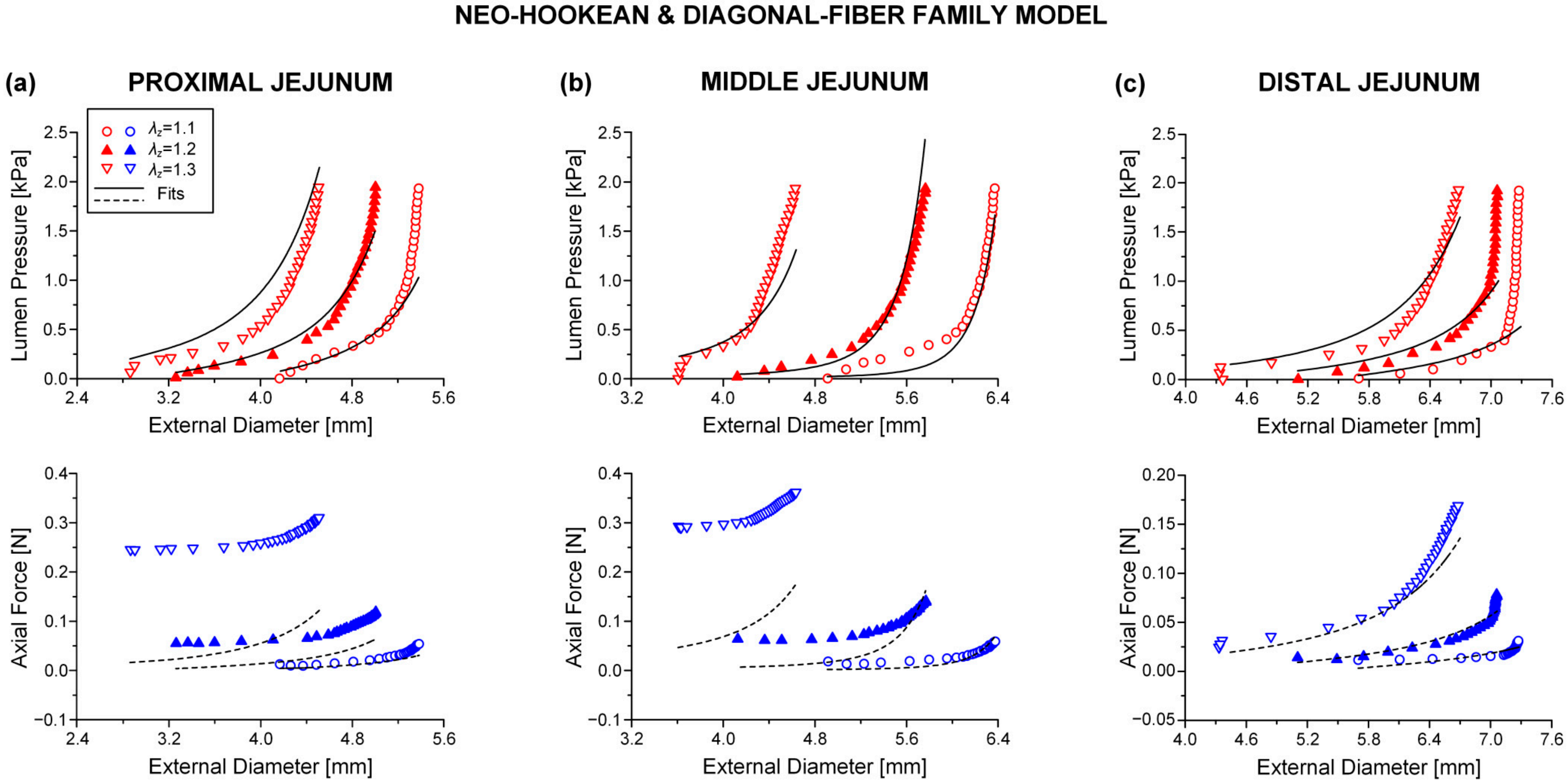

Figure S2. Plot of measured lumen pressure (red color) and axial force (blue color) vs. external diameter data at three different fixed axial stretches λ_z ={1.1, 1.2, 1.3} for a characteristic specimen from the proximal, middle, and distal jejunum and fits (solid lines for lumen pressure and dashed lines for axial force) calculated using the neo-Hookean and diagonal-fiber family microstructure-based model with the following best-fit model parameters for the (**a**) proximal jejunum: $\mu = 6.3 \times 10^{-12}$ kPa, $k_1^d = 4.982$ kPa, $k_2^d = 6.803$, $\alpha_0 = 0.636$ rad, $\varepsilon = 0.493$, $R^2 = 0.688$, $det(\mathbf{R}) = 0.098$, (**b**) middle jejunum: $\mu = 1.5 \times 10^{-13}$ kPa, $k_1^d = 0.187$ kPa, $k_2^d = 7.722$, $\alpha_0 = 0.310$ rad, $\varepsilon = 0.497$, $R^2 = 0.673$, $det(\mathbf{R}) = 0.007$, and (**c**) distal jejunum: $\mu = 3.1 \times 10^{-12}$ kPa, $k_1^d = 2.158$ kPa, $k_2^d = 3.821$, $\alpha_0 = 0.627$ rad, $\varepsilon = 0.363$, $R^2 = 0.801$, $det(\mathbf{R}) = 0.108$. Data are shown every 0.5 mmHg for clarity.

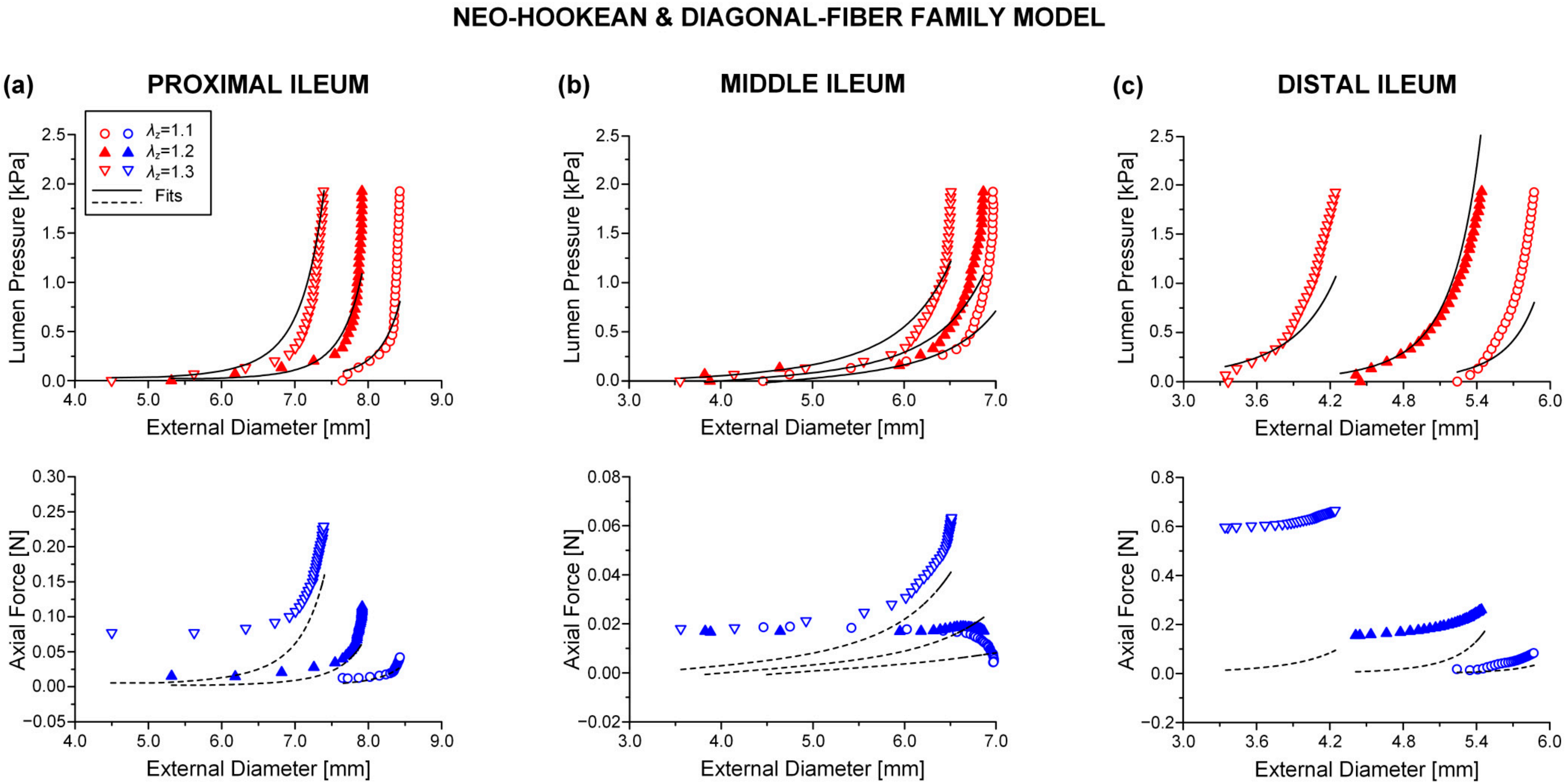

Figure S3. Plot of measured lumen pressure (red color) and axial force (blue color) vs. external diameter data at three different fixed axial stretches λ_z ={1.1, 1.2, 1.3} for a characteristic specimen from the proximal, middle, and distal ileum and fits (solid lines for lumen pressure and dashed lines for axial force) calculated using the neo-Hookean and diagonal-fiber family microstructure-based model with the following best-fit model parameters for the (**a**) proximal ileum: $\mu = 3.5 \times 10^{-9}$ kPa, $k_1^d = 0.110$ kPa, $k_2^d = 4.471$, $\alpha_0 = 0.487$ rad, $\varepsilon = 0.393$, $R^2 = 0.782$, $det(\mathbf{R}) = 0.008$, (**b**) middle ileum: $\mu = 7.6 \times 10^{-18}$ kPa, $k_1^d = 4.800$ kPa, $k_2^d = 12.925$, $\alpha_0 = 0.695$ rad, $\varepsilon = 0.570$, $R^2 = 0.563$, $det(\mathbf{R}) = 0.119$, and (**c**) distal ileum: $\mu = 8.0 \times 10^{-15}$ kPa, $k_1^d = 3.398$ kPa, $k_2^d = 29.514$, $\alpha_0 = 0.666$ rad, $\varepsilon = 0.625$, $R^2 = 0.521$, $det(\mathbf{R}) = 0.066$. Data are shown every 0.5 mmHg for clarity
